# *Foxl2 *functions in sex determination and histogenesis throughout mouse ovary development

**DOI:** 10.1186/1471-213X-9-36

**Published:** 2009-06-18

**Authors:** José Elias Garcia-Ortiz, Emanuele Pelosi, Shakib Omari, Timur Nedorezov, Yulan Piao, Jesse Karmazin, Manuela Uda, Antonio Cao, Steve W Cole, Antonino Forabosco, David Schlessinger, Chris Ottolenghi

**Affiliations:** 1 Laboratory of Genetics, NIA/NIH-IRP, Baltimore, USA; 2 División de Genética, Centro de Investigación Biomédica de Occidente, CMNO-IMSS, Guadalajara, México; 3 Istituto di Neurogenetica e Neurofarmacologia, Consiglio Nazionale delle Ricerche, Cittadella Universitaria di Monserrato, Monserrato, Cagliari, Italy; 4 Department of Medicine, Division of Hematology-Oncology, UCLA School of Medicine, Los Angeles, CA 90095-1678, USA; 5 Unità di Genetica Medica, Università di Modena, Modena, Italy; 6 Current address: UMR-S747 Inserm-Université Paris Descartes, Paris, France

## Abstract

**Background:**

Partial loss of function of the transcription factor *FOXL2 *leads to premature ovarian failure in women. In animal models, *Foxl2 *is required for maintenance, and possibly induction, of female sex determination independently of other critical genes, e.g., *Rspo1*. Here we report expression profiling of mouse ovaries that lack *Foxl2 *alone or in combination with *Wnt4 *or *Kit*/*c-Kit*.

**Results:**

Following *Foxl2 *loss, early testis genes (including *Inhbb, Dhh*, and *Sox9*) and several novel ovarian genes were consistently dysregulated during embryonic development. In the absence of *Foxl2*, expression changes affecting a large fraction of pathways were opposite those observed in *Wnt4*-null ovaries, reinforcing the notion that these genes have complementary actions in ovary development. Loss of one copy of *Foxl2 *revealed strong gene dosage sensitivity, with molecular anomalies that were milder but resembled ovaries lacking both *Foxl2 *alleles. Furthermore, a *Foxl2 *transgene disrupted embryonic testis differentiation and increased the levels of key female markers.

**Conclusion:**

The results, including a comprehensive principal component analysis, 1) support the proposal of dose-dependent *Foxl2 *function and anti-testis action throughout ovary differentiation; and 2) identify candidate genes for roles in sex determination independent of *FOXL2 *(e.g., the transcription factors IRX3 and ZBTB7C) and in the generation of the ovarian reserve downstream of *FOXL2 *(e.g., the cadherin-domain protein CLSTN2 and the sphingomyelin synthase SGMS2). The gene inventory is a first step toward the identification of the full range of pathways with partly autonomous roles in ovary development, and thus provides a framework to analyze the genetic bases of female fertility.

## Background

Menopause, one of the most clear-cut features of female mammalian aging, ensues when the ovarian follicle pool is depleted [1-3]. Hormonal regulation of ovulation from antral follicles is well studied, and many gene mutations and disruptive agents are known to affect oocyte quality and quantity at defined stages of ovary development and maturation. However, the formation and maintenance of the follicle reserve have remained less defined [4-9]. Similarly unclear is the mechanism that connects follicle formation – perhaps the most critical stage of ovary differentiation – to sex determination, the process whereby the sexually bipotential gonad forms either a testis or an ovary [5,10-17].

Acting with the master regulator *SRY*, a large number of genes are currently known to drive testis differentiation in mammals (e.g., [18]). By contrast, although several potent regulatory genes that act in early ovary development have been identified – including those encoding the secreted proteins WNT4 and RSPO1, and the transcription factors, DAX1/NR0B1 and FOXL2 – their relation to now classical hypotheses about the genetic basis of female sex determinantion in mammals [19,20] is still debated, and their mechanism of action is controversial ([17,21-24], and references therein). One striking distinction among them is that the other "early ovarian" genes are mainly expressed in somatic cells of the bipotential gonad in both sexes; but *Foxl2 *is only expressed in females. Furthermore, mutations in *Foxl2 *are notably involved in three well-defined conditions that cover the entire spectrum of ovarian pathology: premature ovarian failure with and without ovarian dysgenesis in humans [25]; blockage of follicle formation with secondary partial ovary-to-testis sex reversal in mice [14,26]; and embryonic sex reversal, sometimes leading to complete XX maleness in goats [27].

We have further shown that in mice the combined loss of *Foxl2 *and *Wnt4 *leads to extensive gonadal XX sex reversal involving all cell lineages [17]. A similar degree of sex reversal was recently obtained in a mouse knockout model for *Rspo1 *[21]. In humans, *RSPO1 *is the only *ovarian *gene known to be associated with complete XX sex reversal [28], and in mice, it is at least partly required for *Wnt4 *expression [21]. In fact, *Foxl2 *and *Rspo1 *are expressed independently of one another throughout fetal life [17,21-23]. Thus, *Foxl2 *and *Rspo1 *regulate distinct female sex determining pathways and redundantly antagonize the action of testis determinants such as the transcription factor SOX9.

Like its regulatory gene *Rspo1 *[21], *Wnt4 *activation in the embryonic ovary is also independent of *Foxl2 *throughout mouse fetal life ([17] and this study). This indicates that studies of *Wnt4 *and *Foxl2 *can provide important complementary information about female sex determination and early ovary differentiation. We have compared ovarian transcriptomes of wild-type mice with mice lacking *Wnt4*, *Foxl2*, or both in the context of comparative analyses of all available microarray datasets for gonadal development and maturation. The results point toward highly specific candidate genes involved in gonadal sexual dimorphism, ovary aging, or both. The putative gene targets were in general consistent with further ovarian transcriptome analyses of ovaries deficient in oocytes (*Kit/c-kit Wv/Wv*), and were validated by corresponding changes in their expression in mouse embryos overexpressing transgenic *Foxl2*.

## Results

### 1. Overview of gonadal development and maturation by principal component analysis: convergence of the maturing ovary to a testis-like profile

To focus on ovary maturation and aging in relation to gonadal development, we compared comprehensive gene expression profiles among a set of 141 microarray gonadal samples, of which 43 come from our study and 98 were reanalyzed from public databases deposited by 4 other laboratories in addition to ours (see Methods and below, section 2). The samples spanned the life history of the mouse ovary and testis from the bipotential gonad to adulthood, including several mouse models of premature ovarian failure. In order to identify major expression profiles associated with distinct genotypes and developmental stages, we used principal component analysis (PCA), an unsupervised clustering method that does not rely on *a priori *class assignment. Essentially, the method first identifies the group of genes with correlated expression profiles that account for the greatest amount of variance in the dataset (the first principal component, PC1), and then sequentially looks for additional co-expression profiles that are not correlated with the first group and account for decreasing amounts of variance (see Methods).

**Figure 1:**
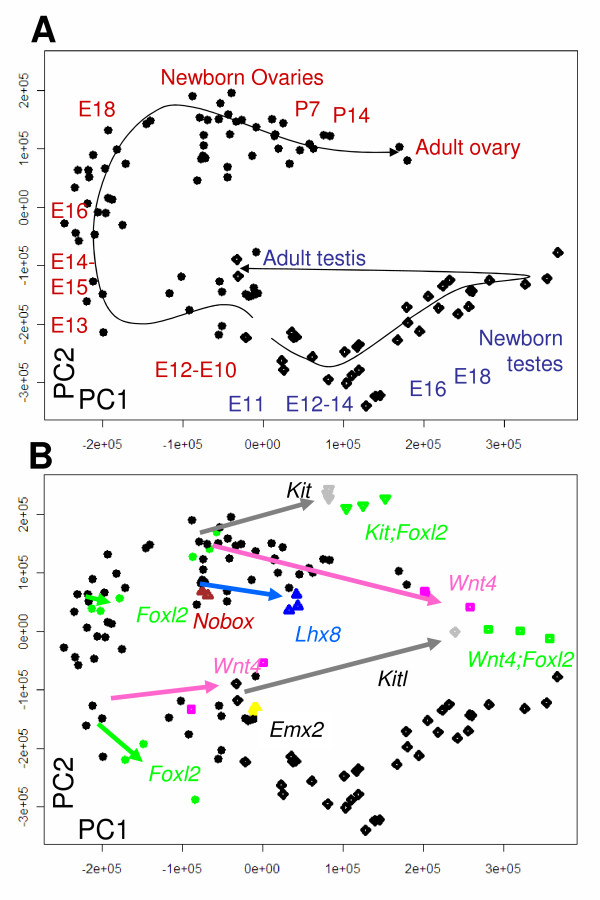
**First two principal components of PCA trained on the expression profiles of 6,455 developmental marker genes**. Several published gonadal microarray datasets ("test dataset") have then been mapped onto the PCA space (our large panel of wild-type and mutant gonads) (x-axis, PC1; y-axis, PC2; PC units, linear transformations of the rank-normalized gene expression intensities). A) Illustration of the trajectories (arrow-headed lines) corresponding to *normal *gonadal development in females (circles) and males (diamonds). B) Trajectories of *mutant *gonads (colored shapes) added to the wild-type panel (i.e., as in A; arrows have the same colors as the corresponding samples).

In Figure 1A, two distinct groups of correlated profiles, represented by the first two principal components (PC1 and PC2), robustly discriminated gonadal samples into appropriate clusters of biological replicates, placing all testis (diamond) and ovary (circle) samples in trajectories according to developmental stages (details about the samples are given in additional files 1A–B and 2A). In Figure 1B, colored symbols are added to show the relative positions of clusters at specific times when particular genes of importance were ablated singly or in combination. The 300 most variable genes were sufficient for good discrimination, but better resolution was obtained with longer lists, i.e., about 6,000 top-ranking markers (Figure 1 and see additional file 2A).

PC1, the first principal component, i.e., the horizontal axis of Figure 1, represented ~40% of the total variance of the developmental gonadal marker genes. In the bipotential gonad, at E10–E12, as expected, sets of genes were close by in ovary and testis. Then, along PC1, developing ovaries first diverged sharply from embryonic testes and subsequently moved back to values that were increasingly similar to newborn testes (E18 to adult ovary, as labeled at the top of Figure 1A). The trend is thus in keeping with morphological indications that some features of follicle maturation involve testis differentiation-like processes (see [16] for a recent review).

**Figure 2:**
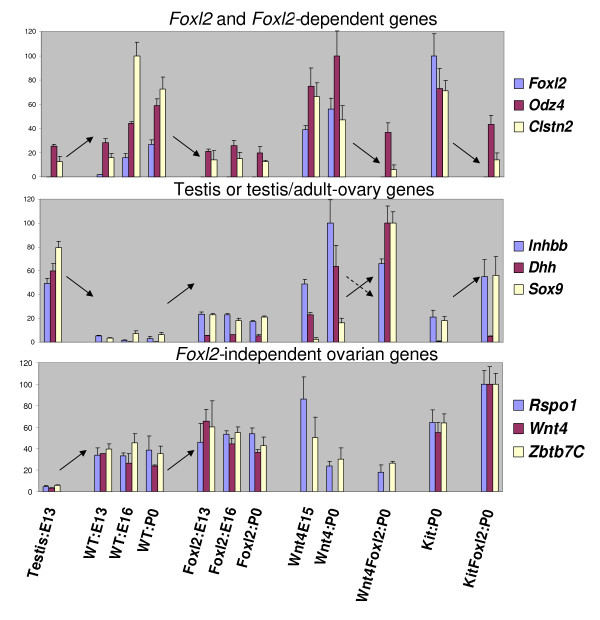
**Real-time PCR data for fetal and newborn gonads of all relevant genotypes**. Top: Ovarian genes that require *Foxl2*; Middle: Testis genes that are derepressed in the absence of *Foxl2*; Bottom: Ovarian genes that are independent of *Foxl2*. Note that several of these genes are differentially regulated in *Foxl2*-null ovaries aged 13.5 dpc. Arrows indicate the relevant changes in expression levels between neighboring conditions (unexplained change involving *Inhbb *as discussed in the text: dotted arrow). For each gene, normalized expression intensities (y-axis) are represented as a fraction of the maximum mean value observed (the latter being set to 100). The x-axis indicates the genotype and stage (E stands for "days post coitum" and P0 for birth, "0 days post natum").

The genes that showed sharp timing differences in their levels of expression along PC1 (see Methods) included those involved in meiotic activity as well as other early ovary somatic cell markers (e.g., *Irx3*, *Fst*, and *Dmc1h*) that had maximum values at the left of the PC1 axis. In addition, many top-scoring genes were involved in steroidogenesis and other early features of testis somatic cell differentiation (e.g., *Dhh, Cyp17a1 *and *Cyp11a1*), with maximum values at the right of the PC1 axis (Figure 1A and see additional file 2B). Furthermore, their PC1 positions in Figure 1B illustrate that *Wnt4*^-/- ^ovaries and, to a lesser extent, *Foxl2*^-/-^, *Kit*^Wv/Wv ^and *Lhx8*^-/- ^ovaries, mapped consistently closer to testis than did age-matched wild-type control ovaries. Also, double mutants showed additive or synergistic effects on "movement toward testis". However, the relative contribution of reduced meiotic activity *vs *increased activity of steroidogenic genes and/or testis-like programs appears to differ in each type of mutant. Indeed, newborn ovaries lacking *Kit, Lhx8 *or *Wnt4 *lie relatively close together along PC1 in spite of their very different composition in germ cell numbers and their highly divergent degrees of sex reversal (see below). Consistent with distinct roles of meiotic and testis genes along the PC1 axis, an adult, germ cell-depleted, *Kitl*-deficient testis sample mapped at the far right of the graph (Figure 2B), i.e., nearer newborn testes than adult wild-type testes (the latter are located in the middle of the graph). Thus, multiple pathways, ranging from control of meiosis to somatic cell sex reversal, are involved in PC1.

In the case of the second axis (PC2) as well, multiple distinct pathways may be involved, but because they appear dissociated by PCA, they are likely different and relatively autonomous from those accounting for PC1. Of interest, PC2 pointed toward many genes involved in follicle formation. For example, newborn ovaries, which are enriched in primordial follicles (e.g., [1,2]), were remarkably clustered at the top of PC2, and several genes involved in early follicle development, i.e., *Lhx8, Figla*, and *Sohlh1 *(cf. [6]) strongly correlated with this axis (see additional file 2C). However, follicle formation is only part of the underpinning of PC2, because some newborn ovary samples (e.g., *Kit *mutants) were also high-scoring even though they are known to be largely devoid of follicles.

Overall, a representative "gonadal developmental transcriptome", trained on pre- and neonatal gonadal samples by PCA, appears to discriminate the range of wild-type gonadal variation throughout life. In addition, PCA suggests that dysgenetic ovaries activate programs that may represent the anticipation of a normal maturation/aging process and which may be a causative factor for associated pathology. However, PCA does not discriminate between possible different contributions to this aging-like process, in particular the effects of meiotic germ cell depletion *vs*. the activation of *bona fide *testis-like pathways in somatic cells. In addition, the expression profiles of some genes with important gonadal functions, including *Foxl2 *and *Sox9 *(see below), correlated only weakly with PCA coordinates. In particular, they both ranked > 100^th ^among genes that were ordered according to their degree of correlation with PC1 using the Focus classifier (see additional file 2C; consistent results were obtained with "predictive analysis of microarray", PAM [29], data not shown). We infer that functional studies of ovary development and maturation may be more incisive if one includes systematic comparisons to *testis *samples in appropriately chosen developmental models. We thus present the molecular characterization of *Foxl2*-null ovaries in the context of other models of ovarian dysgenesis, with special emphasis on the comparison of wild-type ovary and testis differentiation.

#### 2.1. *Foxl2 *knockout ovaries

*Foxl2*^-/- ^knockout ovaries have a histomorphologically normal appearance before birth. To detect molecular changes that may nonetheless predate postnatal anomalies, we sampled ovaries from prenatal and neonatal stages (13.5 and 16.5 days postcoitum, dpc, and birth). These sampling times covered the period of greatest variation in gonadal developmental genes (Figure 1A, above) and could minimize minor stage-specific changes or small shifts in developmental rate that might bias single time points with age-matched samples. To this end, linear contrasts implemented in the Focus software work well [30]: the 20 top-ranking differentially expressed genes (up or down) are listed in the left column of Table 1, and the full lists are given in additional files 3A–B. We also obtained a combined gene list from standard pair-wise analyses, which was highly consistent but much less sensitive (see additional file 3C and Methods). In addition, we sampled for comparison *Wnt4*-null ovaries at 15.5 dpc, i.e., the stage at which partial sex reversal is well started but oocytes are still present and well differentiated.

**Table 1:** *Foxl2*-null ("KO") ovaries relative to wild-type and in various biological contexts.

	**A. Down in *Foxl2 *KO (induced by Foxl2)**			
**rank**	**Single *Foxl2 *KO**	**Compound *Foxl2 *KOs**	**Single *Foxl2 *KO and compound *Foxl2 *KO**	***Foxl2 *single *KO *and *Wnt4 *single KO**

1	*(Foxl2)*	*(Foxl2)*	*(Foxl2)*	*(Foxl2)*
2	*Thbs2*	*Nupr1*	*Ntn4*	*Thbs2*
3	*Peg10*	*Thbs1*	*Thbs1*	***Kitl***
4	*Sdc4*	*Ephx2*	*Adamts16*	*Gstm1*
5	*Fbn2*	*1810010m01rik/ZG-16p*	*Col8a1*	*Cpa2*
6	*9630031f12rik*	*Loc640441*	Clstn2*	*Il13ra2*
7	***Akr1c14***	***Cyp19a1/aromatase***	*Cpa2*	*ENSMUSG00000069372*
8	*Ntn4*	*Dppa3*	*Smad3*	*Peg10*
9	*Cdkn1b*	*Myo1b*	*Slc26a7*	*Tnfrsf19*
10	*Thbs1*	*Ramp1*	*Chst9*	*9630031F12Rik*
11	*E330037m01rik*	*Slc26a7*	*Loc640441*	*Micalcl*
12	*Col8a1*	*Col8a1*	Lrrc4*	*Dync1i1*
13	*Adamts16*	Plxnc1*	*Bcat1*	*Fbn2*
14	*Serpine2*	*Ntn4*	*Itga6*	***Gpc4***
15	Clstn2*	Clstn2*	*Il13ra2*	*Gstm7*
16	***Grip1***	Odz4*	*Nupr1*	*Fbn2*
17	*Speer4b*	*Apoa1*	Plxnc1*	*Upk2*
18	*Rgs2*	*Bcat1*	*Angptl6*	*Cdkn1b*
19	*Cpa2*	Lrrc4*	*Chn2*	***Akr1c14***
20	*Lmo3*	*Pdpn*	Odz4*	*Tmem20*
**B. Up in *Foxl2 *KO (repressed by, or competitive with Foxl2)**				
**rank**	**Single *Foxl2 *KO**	**Compound *Foxl2 *KOs**	**Single *Foxl2 *KO and compound *Foxl2 *KO**	***Foxl2 *single *KO *and *Wnt4 *single KO**
1	*Jakmip1*	*Tesc*	*Adamts8*	*Inhbb*
2	*Slc8a3*	*Pnmt*	*Hsd17b11*	*Fbln2*
3	*B3galt6*	***Dmrt1***	*Fbln2*	***Dhh***
4	*Rps9*	***Sox9***	***Dhh***	*Ndrg2*
5	*2010004m13rik*	*Gm2a*	*2310046k01rik*	*Ptn*
6	*Hemgn*	*BC021891*	*Nr0b1/Dax1*	*Tpm2*
7	*Ndrg1*	*Etv5*	*4632411j06rik*	***Stra6***
8	*Mmp23*	*Mmd2*	*Ifitm7*	*Thbd*
9	*Ptpre*	*Fxyd6*	*Gas7*	*Myl1*
10	*Tnni3*	*Etv5*	*Mcm7*	*Scn5a*
11	***Inhbb***	***Cyp26b1***	*Efhd1*	*Col6a2*
12	*Efhd2*	*Il6st*	*Il6st*	*LOC669875///Tspan15*
13	*Ermap*	*Ecrg4*	*Dsp*	*Adi1*
14	*Adamts8*	*Itm2a*	*Bc019731*	*Adamts8*
15	*Lgals1*	*Fbln2*	*Gm2a*	*Tpm2*
16	*Dmpk*	***Dhh***	*1500015o10rik/Ecrg4*	*Aqp5*
17	*Hsd17b11*	*Bc019731*	*Mmd2*	*Gtl2*
18	*Eraf*	*D3bwg0562e*	*Cdon*	*Dsp*
19	*Wnt11*	*Gpc3*	*2810026p18rik*	*Gna14*
20	*Slc4a1*	*40787*	*Dusp26*	*Filip1*

The twenty top-ranking genes showing significant down- or up-regulation (upper, A, vs lower half, B, of the table, resp.) in *Foxl2*-null ("KO") ovaries relative to wild-type and in various biological contexts. From left to right, top-ranking genes are given for the following conditions: ovaries lacking only *Foxl2 *(aged 13.5 dpc to birth); shared between newborn ovaries lacking *Foxl2 *and *Kit *and those lacking *Foxl2 *and *Wnt4*; shared by all three models (that all involve *Foxl2 *loss); and shared between embryo-fetal ovaries lacking *Foxl2 *and those lacking *Wnt4 *alone [*Foxl2 *ranks at the top because one or more of the conditions under analysis involve its ablation]. Genes known to be expressed in vasculature or germ cells are underlined, neuronal genes have an asterisk, and testis (lower, B) and other genes of interest (upper, A) are in **bold **font.

We first adopted a strategy to identify highly specific (though possibly indirect) targets of *Foxl2*, as follows.

#### 2.2. Candidate genes for a primary role downstream of *Foxl2*: profiling of compound knockout models involving *Foxl2*

We reasoned that primary targets of *Foxl2 *would tend to be differentially expressed in all ovaries lacking *Foxl2 *either alone (from the above analysis) or in combination with the ablation of *Wnt4 *or *Kit*. At birth, these various mouse models show very different morphology, cell composition, and endocrine function, and may thus provide a stringent framework for the identification of *Foxl2*-dependent genes [17]. We compiled lists for genes that were differentially expressed in *Wnt4*^-/-^*Foxl2*^-/- ^and *Kit*^-/-^*Foxl2*^-/- ^double knockout ovaries relative to ovaries lacking *Wnt4 *or *Kit *function but harboring a functional *Foxl2 *allele (see additional file 4A–B, the 20 top genes up- and down-regulated are given in Table 1). We then intersected these lists with lists of genes associated with loss of *Foxl2 *alone (see above). That list comprises 190 and 78 genes that were systematically down or up-regulated in the absence of *Foxl2 *(see additional file 4C; the 20 top genes up and down are given in Table 1, second column from right). These lists thus include highly specific genes that may mediate *Foxl2 *action, as follows.

Inspection of the top genes in the lists (Table 1) indicates that a large fraction of *Foxl2*-dependent genes have no previously assigned role in gonadal development but have been associated with either neuronal or vascular development (as indicated by asterisk or underline, respectvely, in Table 1, column 3 from the left). Some show female-specific upregulation in supporting or steroidogenic cells of embryonic gonads, including a transcription factor, ODZ4, and several plasma transmembrane proteins, including plexin (PLXNC1), the cadherin-domain containing Calsyntenin 2 (CLSTN2), and a leucine-rich repeat protein (LRRC4) (bold font, Table 1). Because some of these genes are involved in brain cortical patterning [31,32], they might conceivably mediate the formation of the ovarian cortico-medullary axis, which is essential for follicle dynamics and reproductive longevity in mammals, and has no histoanatomical counterpart in the testis [33].

Top-ranking down-regulated genes also included *Grip1 *(glutamate receptor interacting protein 1), a nuclear repressor required for estrogen receptor alpha activity, and the gene encoding the AKR1C14 aldo-keto reductase, which can metabolize the most potent natural androgen, dihydrotestosterone [34,35]. *Foxl2*-dependence of these genes during embryonic development (Figure 2) might reflect an early role for *Foxl2 *in regulating sex-specific steroidogenesis, which may be particularly important in higher mammals. Furthermore, starting around 16.5 dpc, upregulation of aromatase/*Cyp19a1 *and the nuclear receptor *Nr5a2/LRH-1*, related to the master regulator of steroidogenesis, *Nr5a1/Steroidogenic factor-1*, was strongly reduced in *Foxl2*-null ovaries relative to controls.

Additional hits further down the lists included sphingomyelin synthase *Sgms2 *(see additional file 4C). It may be a positive regulator of primordial follicle formation and/or maintenance, given that loss of a sphingomyelinase, which performs the reverse metabolic reaction, was reported to enhance primordial follicle maintenance in mice [36]. Another hit, encoding the cytoskeletal protein BICD1 that is related to a maternal fertility factor in *Drosophila*, was enriched in primordial follicles of both oocytes and somatic cells (data from [37,38]). It may complement the action of a paralogue, BICD2, that we have previously shown to be expressed in growing oocytes [39].

Expression levels were sometimes altered starting from 13.5 dpc, as validated by real-time PCR (Figure 2). There the levels in E13 testis, first three bars at the left of each row, are compared to levels in the ovary collected at various developmental stages in wild-type and mutant mice; the corresponding microarray data (see additional file 5) are concordant in every case. Their relevance as candidate *Foxl2 *targets was further substantiated by experiments in which an added *Foxl2 *transgene was provided to wild-type mice (see below).

The comparison of ovaries lacking *Foxl2 *alone or in combination with either of two other genes therefore prioritizes candidate target genes that show sensitivity to *Foxl2 *loss in the independent mouse models. Candidate *Foxl2 *targets may also be inferred by their response to multiple ovarian regulatory pathways, as follows.

#### 2.3. Candidate *Foxl2 *target genes that respond to *Wnt4 *or other ovarian pathways

**Figure 3:**
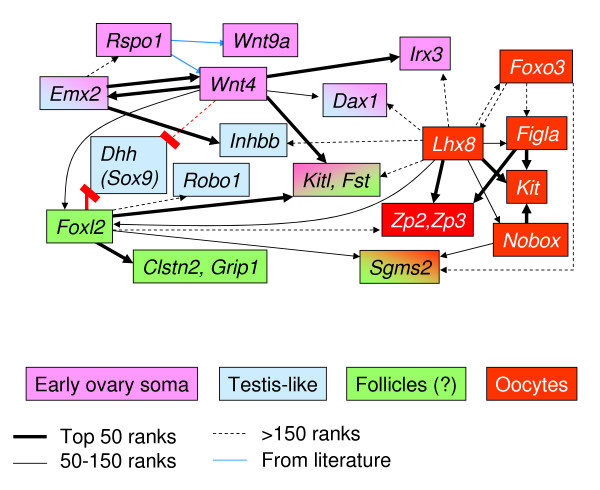
**Putative interactions among developmental genes inferred from the comparison of several knockout models of ovarian dysgenesis**. Arrows indicate positive interactions. Most cases of competitive or antagonistic interactions are not represented, because all genes except *Sox9 *and *Dhh *can be connected by (positive) arrows with at least an ovarian gene. *Sox9 *and *Dhh *are not induced by any of the ovarian genes that have been functionally tested, and are antagonized specifically by *Foxl2 *and *Wnt4*. Color coding is purely indicative of overall gonadal expression and/or function (legend is at the bottom). Note that inferences regarding direct or indirect regulatory biochemical interactions cannot be derived from this type of analysis: arrows only indicate the apparent requirement of a gene (to which the arrow points) for the function of another gene (from which the arrow originates) as assessed in knockout mice. This identifies a putative target-regulator connection. Line thickness indicates the degree of statistical association between the putative target gene and the putative regulator.

We cross-compared all mouse models of ovarian dysgenesis that have been studied by microarray expression profiling to date. They include knockouts of the somatic genes *Emx2 *and *Wnt4*, and the oocyte genes *Lhx8, Nobox, Foxo3 *and *Figla1 *[40-46]. We reanalyzed the respective datasets compared to their respective controls (see Methods and additional files 6A–J). As expected for genes that are all required for fetal and newborn ovary development, the resulting lists of differentially expressed genes indicated similar alterations, reflecting synergistic effects. In particular, several well-known ovarian markers were comparably changed. Inferred putative interactions are represented in Figure 3, along with others that involve important novel candidate targets of *Foxl2 *(*Clstn2, Grip1*, and *Sgms2*, see above). *Arrows *indicate gene dependencies in terms of ranking by statistical significance in the microarray analyses; thus, the arrowhead points to gene(s) that score among the top hits (i.e., for *thick *arrows, ranks 1 through 50) in the list of genes affected by the gene from which the arrow originates. [Some gene dependencies that have been shown to be strong in *Rspo1*-null ovaries, for which microarray data are not available, are indicated with blue arrows.] For example, *Wnt4 *and *Emx2 *scored among the top hits in the respective lists of *Emx2*- and *Wnt4*-dependent genes, respectively, consistent with previous reports [40,41]. Similarly, zona pellucida genes *Zp2 *and *Zp3 *were identified among the genes most strongly down-regulated in the absence of *Lhx8, Nobox *and *Figla*. In addition, *thin *and *broken-line *arrows indicate progressively lower degrees of dependence (i.e., rankings arbitrarily set at 50–150 and > 150, resp.); for instance, intermediate (thin arrow) dependencies include that of *Dax1/Nr0b1 *for *Wnt4*, a dependence that is known to be only partial [47].

Overall, Figure 3 shows that in spite of a widespread network of positive correlations among well-known ovarian genes, they are affected to a different degree in distinct pathological conditions, with a quite clear-cut separation of the main candidate targets of somatic genes *Emx2/Rspo1/Wnt4/Foxl2 *from those of oocyte genes *Lhx8/Nobox/Figla*/*Foxo3 *(green/pink vs red boxes, resp., in Figure 3; different colors are used for *Foxl2 *and *Wnt4*-related genes because of their partial independence, see below). This is consistent with the proposal that somatic cells and oocytes may act on largely distinct though complementary pathways to promote ovary development. The relation of these genes to testis-like markers (blue boxes in Figure 3) was more complex (see below).

We then extended the analysis from known markers to the complete gene lists obtained by microarray profiling, and thus determined their degree of overlap by chi-square (Table 2 and see Methods). As expected, most lists were positively correlated with one another (i.e., genes up- or down-regulated in the absence of one ovarian gene tended to change in the same direction in the absence of the others; see additional files 6A–H). Nevertheless, one exceptional trend was notable: the lists of genes that depend on *Foxl2 *and those dependent on *Wnt4 *or *Emx2 *were negatively correlated. In other words, genes that were up-regulated in the absence of *Wnt4 *or *Emx2 *were preferentially down-regulated in the absence of *Foxl2*, and *vice versa *(p < 0.001, chi-square; see additional file 7). In particular, important developmental genes that were up in *Foxl2*-null ovaries and down in *Wnt4*-null ovaries included known or likely *Wnt4*-dependent genes in the ovary [*Nr0b1/Dax1, Amhr2*, and *Podxl; *the last of which is also a candidate *Wnt4 *target in the kidney (see additional file 7D and data not shown)].

**Table 2:** Degree of concordance between lists of differentially expressed genes in several models of ovarian dysgenesis.

	***Emx2 *KO**		***Wnt4 *KO**		***Foxl2 *KO**	
	Concordant	Discordant	Concordant	Discordant	Concordant	Discordant
***Emx2 *KO**	-	-	**1643**	**635**	(716)	(1292)
***Wnt4 *KO**	-	-	-	-	(610)	(1628)
***Lhx8 *KO**	**1295**	**789**	**1223**	**747**	**1491**	**452**
***Nobox *KO**	**345**	**256**	312	287	**428**	**163**
***Foxo3 *KO**	**500**	**346**	396	347	**530**	**257**
	***Nobox *KO**		***Foxo3 *KO**		*Figla *KO (*)	
	Concordant	Discordant	Concordant	Discordant	Concordant	Discordant
***Lhx8 *KO**	**1166**	**86**	**764**	**495**	**69**	**5**
***Nobox *KO**	-	-	**266**	**69**	**39**	**2**
***Foxo3 *KO**	-	-	-	-	**40**	**3**

Concordance or discordance measured as the number of genes that showed the same or opposite directions of change pairwise comparisons of distinct knockout ("KO") models. The upper part of the table compares somatic genes among themselves and with oocyte genes, the lower part compares oocyte genes (the smaller list reported for *Figla*-null ovaries is indicated by *; see Methods). **Bold **font or underline indicate positive or negative correlations among lists, resp.

These differences were reflected in the relative distribution of several pathways listed in the Gene Ontology and KEGG databases (at FDR 10%; Table 3 and see additional file 8). In particular, glucose metabolism and protein synthesis were strongly enriched in *Foxl2*-null ovaries but depleted in *Wnt4*-null ovaries, respectively, whereas cell-cell interactions and neuronal-like pathways were depleted in *Foxl2*-null ovaries but enriched in the absence of *Wnt4*. Consistent with reduced cell-cell signaling, loss of *Foxl2 *apparently led to reduced cell migratory activities, as inferred from the down-regulation of MAPK and several cancer-related pathways that are presumably involved in ovarian tissue remodeling, as well as apoptotic factors that may have homeostatic actions in these processes. This is consistent with the notion that *Foxl2 *is a critical determinant of ovary histogenesis, notably follicle formation.

**Table 3:** KEGG analysis on gene lists for *Foxl2*-null or *Wnt4*-null fetal ovaries.

	***Foxl2 *null-depleted**		***Foxl2 *null-enriched**		
**Pathway**	**N**	**Proportion**	**N**	**Proportion**	**p-value**
Ubiquitin mediated proteolysis	(42)	(0,06)	11	0,02	0,00003
Insulin signaling pathway	(35)	(0,05)	8	0,01	0,00004
ErbB signaling pathway	(28)	(0,04)	6	0,01	0,00018
MAPK signaling pathway	(58)	(0,09)	26	0,04	0,00067
Axon guidance	(40)	(0,06)	15	0,02	0,00132
Prostate cancer	(22)	(0,03)	5	0,01	0,00146
Colorectal cancer	(27)	(0,04)	8	0,01	0,00179
Long-term potentiation	(19)	(0,03)	4	0,01	0,00252
Adherens junction	(24)	(0,04)	7	0,01	0,0032
Glioma	(19)	(0,03)	5	0,01	0,00644
Apoptosis	(15)	(0,02)	3	0,00	0,0074
Wnt signaling pathway	(41)	(0,06)	19	0,03	0,00773
Alanine and aspartate metabolism	0	0,00	(8)	(0,01)	0,00318
One carbon pool by folate	2	0,00	(13)	(0,02)	0,00333
Glyoxylate and dicarboxylate metabolism	0	0,00	(9)	(0,01)	0,00154
Glycolysis/Gluconeogenesis	3	0,00	(19)	(0,03)	0,00035
Oxidative phosphorylation	7	0,01	(33)	(0,05)	0,00001
Ribosome (protein synthesis)	3	0,00	(79)	(0,13)	0
					
	***Wnt4 *null-depleted**		***Wnt4 *null-enriched**		
**Pathway**	**N**	**Proportion**	**N**	**Proportion**	**p-value**
Ribosome (protein synthesis)	(55)	(0,09)	7	0,01	0
Oxidative phosphorylation	(51)	(0,08)	7	0,01	0
Glycolysis/Gluconeogenesis	(18)	(0,03)	3	0,00	0,00008
Fructose and mannose metabolism	(15)	(0,02)	4	0,01	0,00194
Calcium signaling pathway	16	0,03	(45)	(0,06)	0,00548
Neuroactive ligand-receptor interaction	19	0,03	(52)	(0,07)	0,00441
Leukocyte transendothelial migration	9	0,01	(33)	(0,04)	0,00396
Cell adhesion molecules (CAMs)	7	0,01	(31)	(0,04)	0,00141
Cytokine-cytokine receptor interaction	18	0,03	(54)	(0,07)	0,00135
Natural killer cell mediated cytotoxicity	9	0,01	(36)	(0,05)	0,00115
Complement and coagulation cascades	2	0,00	(19)	(0,02)	0,00131
ECM-receptor interaction	9	0,01	(37)	(0,05)	0,00076
Focal adhesion	23	0,04	(70)	(0,09)	0,00014

Columns from left to right: name of the pathway, number and proportion of genes that are down- or up-regulated in the respective knockout model, and corresponding p-value. All the pathways with FDR < 10% are shown. Underlines indicate pathways that are enriched in the corresponding columns.

Of interest, *Foxl2 *loss also affected *Wnt*-signaling. Global alterations associated with oocyte genes were much less pronounced (none scored at the 10% FDR threshold). Consistent with a complementary action of oocytes and somatic cells, loss of oocyte genes led to a mixture of *Foxl2*- and *Wnt4*-associated features (not shown).

The results thus support our previous findings that *Foxl2 *and *Wnt4 *act independently during ovary differentiation, and further substantiate the notion that they may partly antagonize each other and/or regulate mutually competitive pathways. Nevertheless, some known gonadal genes were top-ranking candidate targets of both *Foxl2 *and *Wnt4*, including *Kitl *and *Fst *(pink-green color in Figure 3 and see additional file 1D). Novel genes of potential interest that were strongly down-regulated in ovaries lacking *Foxl2 *or *Wnt4*, and thus responsive to the sum of their actions, included *Gpc4 *and *Akr1c14 *(see additional file 7A and see Discussion). Additional hits included several transcription factors, e.g., *Msx1, Grip1, Tcf4*, and *Foxp1 *(see additional file 7A). Although they scored relatively low, these genes were up-regulated in *Foxl2*-transgenic mouse embryos (see below). Thus all of these genes, many of them expressed in somatic cells, now become candidates for a *Foxl2*-dependent role in coordinating ovarian histogenesis and/or steroidogenesis.

#### 2.4. Anti-testis roles of *Foxl2 *and *Wnt4*, and of other possible regulatory genes

We next focused on genes that were significantly up-regulated in ovaries lacking *Foxl2 *alone or in combination with *Kit *or *Wnt4*. They included both ovarian and testis genes. The ovarian genes included *Nr0b1/Dax1, Wnt4 *and *Rspo1 *as well as novel candidates, such as *Zbtb7c; *the testis genes included *Sox9, Dhh, Dmrt1*, and *Cyp26b1 *(Table 1, Figure 2, and see additional file 3 and 6). It is notable that these and other top-scoring ovarian and testis genes have important roles in sex determination and/or early sexual dimorphism (reviewed by, e.g., [18]). Thus, in the absence of *Foxl2*, the exacerbation of *Foxl2*-independent ovarian pathways(s) coincided with the partial de-repression of testis-like genes. This started during embryonic development, even in single knockout ovaries that are morphologically normal until birth (see Discussion). Similarly, and consistent with much earlier onset of morphological anomalies, *Wnt4*-null single knockout ovaries showed de-repression of some testis genes, e.g., *Dhh *and *Inhbb*; and although *Foxl2 *was not up-regulated in these dysgenetic ovaries by whole-organ microarray (see additional file 5), its levels remained high compared to other ovarian genes and were increased by real-time PCR (Figure 2, and see below). In addition, FOXL2 protein expression was strongly detected in the persisting cord-like and follicle-like structures (data not shown).

The expression levels of some early testis-like genes showed opposite directions of change in the absence of distinct ovarian genes. In particular, *Inhbb *was up in fetal ovaries lacking either *Wnt4 *or *Foxl2*, but down in ovaries lacking the oocyte gene *Lhx8 *and in the most masculinized phenotype that we have studied, i.e., *Wnt4*^-/-^*Foxl2*^-/- ^double knockout newborn ovaries (Figures 2 and 3). Similarly, *Robo1*, which is up-regulated in *Wnt4*-null ovaries ([43] and see additional file 6B), showed reduced levels in ovaries lacking *Foxl2 *(Figure 3 and see additional file 3A). Therefore, regulation of these testis-like genes, and their impact on the ovarian phenotype, may depend on the conditional interactions of multiple ovarian factors.

Similar to loss of *Foxl2 *or *Wnt4*, loss of *Lhx8 *led to up-regulation of a few genes that are normally expressed in testes (e.g., *Stra6 *see additional file 6F). However, this effect did not extend to genes known to be involved in testis sex determination, suggesting that *Foxl2*- and *Wnt4*-null ovaries are better suited to identify novel anti-testis genes.

To identify novel candidate anti-testis genes with a "master" regulatory role comparable to *Foxl2 *or *Wnt4*, we used a simple statistical approach (Methods) that detects genes that are both testis-depleted and up- or partially down-regulated in the dysgenetic ovary relative to the wild-type ovary. The gene candidates for "independent anti-testis" gene action in the *Foxl2*-null or *Wnt4*-null ovaries are listed in additional files 9A–C, with the top-ranking hits given in Table 4. As expected as an internal control, consistent with their relative independence [17], *Wnt4 *was top-ranking in *Foxl2*-null ovaries, and *Foxl2 *in *Wnt4*-null ovaries. In addition, the overlap between the two lists detected *Rspo1 *[21] and numerous oocyte genes as female-enriched and independent of both *Wnt4 *and *Foxl2 *[the latter may again reflect the overall independence of oocytes from loss of either *Wnt4 *(before birth) or *Foxl2*]. In addition, several other genes were expressed in somatic cells, i.e., *Runx1, Irx3*, a *Foxl2*-antisense transcript, *Wnt9a*, and *Zbtb7c*. The relative levels of all of them were validated by multiple microarray probe sets and/or by real-time PCR (as well as in studies of *Foxl2 *transgenic mice, below). These genes may thus have a role in autonomous pathways capable of antagonizing testis differentiation independently of *Foxl2 *and *Wnt4*.

**Table 4:** The thirty top-ranking genes for an action independent of *Foxl2 *and *Wnt4*.

**Rank**	**GeneId**	**Gene Description**
1	*Xist*	Inactive × specific transcripts
2	*Tspan33*	Tetraspanin 33
3	*Runx1*	Runt related transcription factor 1
4	*Kctd14*	Potassium channel tetramerisation domain containing 14
5	*Tktl1*	Transketolase-like 1
6	*Glt8d4*	Glycosyltransferase 8 domain containing 4
7	*Rspo1*	R-spondin homolog (Xenopus laevis)
8	*Foxl2os*	Foxl2os
9	*Lrba*	LPS-responsive beige-like anchor
10	*Irx3*	Iroquois related homeobox 3 (Drosophila)
11	*Sycp3*	Synaptonemal complex protein 3
12	*Podxl*	Podocalyxin-like
13	*Smc1b*	Structural maintenance of chromosomes 1B
14	*Fndc5*	Fibronectin type III domain containing 5
15	*2010004M13*	RIKEN cDNA 2010004M13 gene
16	*Gm1564*	Gene model 1564, (NCBI)
17	*Lzts1*	Leucine zipper putative tumor suppressor 1
18	*Taf7l*	TAF7-like RNA polymerase II, TATA box binding protein (TBP)-associated factor
19	*1700007E06*	RIKEN cDNA 1700007E06 gene
20	*Zbtb7c*	Zinc finger and BTB domain containing 7C
21	*Emx2*	Empty spiracles homolog 2 (Drosophila)
22	*Gdpd2*	Glycerophosphodiester phosphodiesterase domain containing 2
23	*Ndufa1*	NADH dehydrogenase (ubiquinone) 1 alpha subcomplex, 1
24	*GM1140*	Gene model 1140, (NCBI)
25	*Lrrn1*	Leucine rich repeat protein 1, neuronal
26	*Atp1b1*	ATPase, Na+/K+ transporting, beta 1 polypeptide
27	*Fst*	Follistatin
28	*Myh8*	Myosin, heavy polypeptide 8, skeletal muscle, perinatal
29	*Dmrtc1c*	Doublesex and mab-3 related domain, member 1C
30	*Rgs2*	Regulator of G-protein signaling 2

This list includes candidate genes for an autonomous anti-testis role relative to both *Foxl2 *and *Wnt4*.

### 3. *Foxl2 *transgenic mice

**Figure 4:**
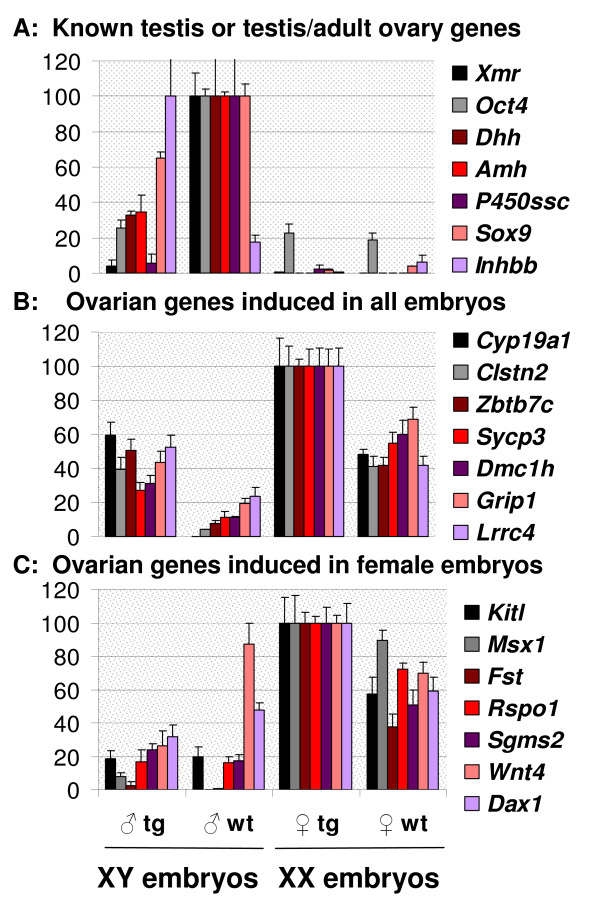
**Expression levels of several gonadal genes in 13.5 dpc gonads from *Foxl2 *transgenic embryos compared to littermates**. A: genes known for a role in early testis differentiation, some of which are also expressed in the adult ovary; B: novel or known ovarian genes that are induced by *Foxl2 *in XY and XX gonads; C: novel or known ovarian genes that are induced by *Foxl2 *in XX but not XY gonads.

We used *Foxl2*-transgenics to see if candidate *Foxl2 *targets inferred from the microarray analyses presented above were indeed responsive to Foxl2 (Figure 4A, B and 4C; there the levels of individual genes are compared in gonads from wild-type males, wild-type females, transgenic males, and transgenic females). As expected, *Foxl2 *expression was absent or at background levels in gonads from XY wildtype embryos. But in XY transgenic embryos, gonadal levels of *Foxl2 *were similar to wildtype ovaries, and XX transgenic ovaries showed even stronger expression (nearly 2-fold, see additional file 10). This correlated with sex-specific effects, as follows.

All female markers normally up-regulated during early ovarian differentiation, whether sexually dimorphic or not, were expressed at consistently higher levels in 13.5 dpc *Foxl2*-transgenic ovaries relative to wild-type littermates (21 genes tested, Figures 4B and 4C and see additional file 11). That included *Rspo1*, *Fst*, *Wnt4*, *Dax1/Nr0b1 *and *Zbtb7c*. In XX embryos, up-regulation overtly extended to the meiotic markers *Sycp3 *and *Dmc1h*. The status of *Foxl2 *candidate targets inferred from the microarray analysis of *Foxl2 *knockout mice is thereby validated, and there are hints of feed-forward interactions with *Foxl2*-independent genes, such as *Wnt4*, *Rspo1 *and *Zbtb7c *(see Discussion).

Furthermore, several ovarian genes were up-regulated to various extents in 13.5 dpc transgenic XY gonads, which contained severely disorganized seminiferous tubules [17]. These changes are consistent with the induction of a limited degree of male-to-female sex reversal in *Foxl2 *transgenics. Among the statistically significant genes were some expressed in meiotic germ cells (*Sycp3 *and *Dmc1h*) and others in somatic cells, i.e., *Zbtb7c, Clstn2, Grip1, Lrrc4 *and aromatase (*Cyp19a1*). In particular, induction of aromatase and *Clstn2 *in *Foxl2 *transgenic XY gonads was remarkably strong relative to testes (Figure 4B). This is in agreement with previous *in vitro *studies indicating that *Foxl2 *can transactivate the aromatase promoter (e.g., [48,49]). Conversely, 13.5 dpc *Foxl2*-transgenic XY gonads showed reduced expression of the male sex determining genes *Sox9 *and *Dhh*. In addition, strong repression was seen for downstream embryonic testis-specific genes of endocrine relevance (e.g., the antimullerian hormone, *Amh*, and *Cyp11a1/P450ssc*, which were reduced 3- and 18-fold, respectively). Repression was also strikingly pronounced for the two male germ cell markers tested, *Xmr *and *Oct4*, which were reduced 26- and 4-fold, respectively (Figure 4A).

A few results were unanticipated; e.g., the *Foxl2 *transgene down-regulated the expression of *Wnt4 *and *Dax1/Nr0b1 *while increasing expression of *Inhbb*. These unanticipated effects may result from an exacerbation of the inferred partial antagonism between *Foxl2 *and *Wnt4*, and possibly relate to the complex responses shown by *Inhbb *attendant on the loss of various ovary genes alone or in combination (see above). In any case, the preponderance of the data provide independent support for an early anti-testis action of *Foxl2 *– whether by delaying development of XY gonads or by promoting male-to-female sex reversal. Most important, the *Foxl2*-mediated disruptive effects, including the suppression of *Wnt4*, were confined to XY embryos. The notion of a specific and early sexually dimorphic role for *Foxl2 *is thereby supported.

### 4. Gene dosage effects in *Foxl2*^+/- ^heterozygous ovaries

Loss of a single copy of *Foxl2 *might represent a model for the heterozygous *FOXL2 *mutations that affect patients. We looked at the extent to which heterozygotes expressed molecular anomalies like those seen in *Foxl2*-null ovaries. We focused on two stages, 16.5 dpc and 7 dpn (days post-natum), because the corresponding *Foxl2*-null ovaries showed well-differentiated morphological states that were either normal or well engaged in abnormal development.

In order to identify molecular anomalies associated with heterozygous loss of function, we generated oocyte- *vs *somatic cell gene lists enriched in fetal vs postnatal ovaries of heterozygous mice. The lists were based on published microarray data, supplemented by a list of the probes for known testis determining genes (Methods and see additional file 12). We then evaluated differential enrichment in these lists by a sensitive algorithm that detects non-random distributions in pair-wise comparisons (Gene Set Enrichment Analysis, or GSEA, [50]; all these results are presented in additional file 13; see Methods).

We first tested testis-determining genes (including *Dmrt1, Sox9, Dhh, Fgf9*, and *Sf1/Nr5a1*), which, as a group, were significantly up-regulated in 16.5 dpc *Foxl2*^+/- ^heterozygous ovaries relative to wild-type (p < 0.03, GSEA), with levels that were comparable to those in *Foxl2*-null ovaries (p = 0.20, GSEA). These findings raise the possibility that loss of a single allele of *Foxl2 *may be sufficient for a significant though weak derepression of testis genes in the fetal mouse ovary. Nevertheless, this effect appeared to be transient. Indeed, in 7 dpn *Foxl2*^+/- ^heterozygous ovaries, the microarrays evinced no significant trend toward the expression of testis markers (p > 0.2, GSEA). This contrasted with Foxl2-null ovaries, which, consistent with our previous studies showing delayed postnatal oocyte growth and partial sex reversal (see above), showed a significant enrichment of testis determining genes at 7 dpn (p = 0.02, GSEA).

Consistent with ongoing sex reversal, 7 dpn *Foxl2*-null ovaries also showed a sharp extensive repression of postnatal oocyte and somatic follicle cell markers compared to both *Foxl2*^+/- ^heterozygous and wild-type ovaries (p < 0.001, GSEA). Compared to *Foxl2*-null ovaries, *Foxl2*^+/- ^heterozygous ovaries exhibited normal expression of oocyte growth genes but showed clear dosage effects for somatic follicle markers. The latter were inhibited almost as much in heterozygotic ovaries as in *Foxl2*-null ovaries compared to wild-type (p < 0.001, GSEA). Furthermore, as expected for a dosage-related response, the inhibition in heterozygous ovaries was less strong than in *Foxl2*-null ovaries (p < 0.001). Top-scoring genes were often known or likely to be involved in early steps of follicle growth, i.e., *Inhba, Inhbb, Cyp11a1/P450ssc, Cyp17a1, Nr5a2 *(see additional file 14), as well as novel genes with a likely role in somatic cells, e.g., *Odz4 *(see above). Several of these genes are known to be involved in steroidogenesis. Thus, in the absence of one *Foxl2 *allele, strong dosage effects in somatic follicle growth genes contrasted sharply with the normal expression of oocyte growth genes.

In addition, in 16.5 dpc and 7 dpn *Foxl2*^+/- ^heterozygous ovaries, we found significant up-regulation of markers for *fetal *ovary development, independent of whether they were germline- or somatic cell-enriched (e.g. *Figla, Sohlh1, Sycp3*, *vs Rspo1, Irx3, Gng13*, p < 0.001 and p < 0.01, resp., GSEA). This suggests that *Foxl2*^+/- ^heterozygous ovaries undergo a developmental delay starting in fetal life.

## Discussion

We have analyzed mouse ovary development and maturation based on comparative gene expression profiling of wild-type stages and pathological conditions. First, we used an unsupervised clustering algorithm, PCA, to show that gonadal developmental genes can discriminate stages of ovarian differentiation and maturation throughout life. However, sexual dimorphism was resolved only partly, i.e., conditional on developmental time. Thus, as a second, supervised approach, we focused on *Foxl2*-null ovaries.

Cellular models are currently lacking for both sex determination and follicle formation. Whole-organ studies provide an alternative route to investigate underlying developmental processes, which involve complex interactions of cell types. However, whole-organ studies are particularly sensitive to biological confounds such as overt changes in cell-type composition. To circumvent such effects, we compared conditions that were associated with highly divergent ovarian phenotypes but shared a common genotype, i.e., loss of *Foxl2 *or *Wnt4 *alone or *Foxl2 *in combination with *Kit *or *Wnt4 *(Section 2.2). Indeed, largely distinct cell types are affected in the null ovaries that lack *Foxl2*, *Kit *or *Wnt4 *(i.e., somatic supporting cells, germ cells, and stroma cells, resp.). It is thus unlikely that secondary and conditional targets of *Foxl2 *would be similarly affected by chance in the very different phenotypes associated with these three models of ovarian dysgenesis. Thus, the lists resulting from a series of distinct dysgenetic ovary models involving loss of *Foxl2 *should be enriched in primary targets of this gene. Similar considerations led us to search for common putative targets of *Foxl2 *and *Wnt4*, as their deletion induces somewhat complementary phenotypes (Section 2.3). The analysis enables us to suggest novel genetic interactions and ovarian genes that are either candidate targets or act independently of *Foxl2 *and *Wnt4*. Results were validates by *Foxl2 *overexpression studies and the analysis was extended to *Foxl2*^+/- ^heterozygotes (Sections 2.4–4).

The data lead to four main inferences.

### 1) Partial convergence of somatic transcriptomes in maturing ovary and testis

One inference from the present analysis is that by unsupervised principal component analysis (PCA), meiotic activity and some testis-related pathways (which include genes such as *Dhh *and *Cyp11a1*) may be dissociated from other pathways that include follicle formation (which include *Lhx8 *and *Figla*), as they map along two different axes (PC1 vs PC2). This suggests that two major pathways, one more closely related to germ cell differentiation and the other to somatic cell histogenesis, may act autonomously during gonadal development. This is consistent with some current theories on the mechanisms of sexual dimorphism; but the range of autonomous (groups of) pathways may be greater than two, as indicated by our supervised analyses (see below)

A second inference from PCA is that a large fraction of the mouse ovarian developmental transcriptome becomes progressively similar to testis after birth. As shown in Figure 1A, PCA discriminates fetal and newborn ovaries from wild-type adult gonads, which cluster with fetal-newborn testes along PC1. This supports accumulating evidence for a link between ovary maturation and a predisposition of this organ to form testis-like tissues [16]. We found that the shift toward testes was accelerated in several knockout or mutant ovary models compared to age-matched wild-type ovaries (Figure 1B). However, PCA did not discriminate between contributions to this pattern from the loss of meiotic activity or from the activation of testis-like pathways expressed in somatic cells. In addition, some critical gonadal genes correlated only poorly with PCA coordinates. *Foxl2 *and *Sox9 *were notable examples. PCA failed to detect their standing as the current best markers for wild-type female and male supporting cells, exemplified by their striking pattern of mutually exclusive expression in a wide range of different conditions involving partial sex-reversal [14,17,51]. Rather than conferring variable degrees of predisposition to sex reversal, *Foxl2 *and *Sox9 *seem to act as all-or-none switches, and their mutual antagonism may be determinative in sex determination [16]. We further tested this proposal by supervised analyses of the *Foxl2*-null ovary transcriptome.

### 2) Specific gene candidate targets of *Foxl2 *and comparison of ovaries lacking *Foxl2 vs *other early ovarian factors

By comparing single and compound knockout ovaries lacking *Foxl2*, we have identified and ranked 149 candidate genes for a specific role downstream of *Foxl2 *(top gene list in Table 1, second column from right, and see additional file 4 for the full list). KEGG pathway analysis indicated that in *Foxl2*-null fetal ovaries, neuronal-like pathways, migratory activity and cell-cell interactions were reduced, whereas glucose metabolism and protein synthesis were increased (Table 3 and see additional file 8). Of note, *Wnt4*-null ovaries showed a largely inverse metabolic profile, and we observed a significant negative correlation between the inferred candidate target genes of *Wnt4 *and *Foxl2 *(Table 2 and Figure 3). This contrasts with results when several other ovarian genes are ablated. Those mouse models show largely positive correlations, though the strength of dependence of the shared candidate targets varies, notably between somatic genes and oocyte genes (Table 2 and Figure 3; see Results). Thus, the widely divergent molecular anomalies observed in *Wnt4 *and *Foxl2*-null fetal ovaries provide an entry point to the mechanisms underlying the partly autonomous, complementary roles of these genes in ovary development.

Consistent with KEGG results, several genes that are known to be expressed by neurons and vascular cells in other systems, e.g., *Calstn2, Odz4 *and *Lrrc4*, were among the most sensitive candidate *Foxl2 *targets (Table 1). In addition, some steroidogenesis-related genes (*Grip1 *and *Akr1c14*) were also affected very early by loss of *Foxl2*. This early effect is striking considering that in the mouse ovary, active steroidogenesis occurs postnatally. Steroidogenesis is completely repressed in postnatal *Foxl2*-null mouse ovaries [26] and steroidogenic genes are strongly affected in *Foxl2 *transgenic mouse embryos (discussed below). Some additional *Foxl2*-dependent genes, expressed in both somatic cells and oocytes, may be associated with novel ovarian functions, including *Sgms2 *and *Bicd1 *(see Results).

Comparison of the positive candidate targets shared by *Foxl2 *and *Wnt4 *pointed to additional genes that may integrate the outputs of the respective largely independent pathways (Table 1 and see additional file 8). They include the genes encoding the well-known Kit ligand, KITL, the *Wnt4*-effector Follistatin, the proteoglycan GPC4, and several other candidate genes for novel roles in ovary development, i.e., *Msx1, Grip1, Tcf4*, and *Foxp1 *(bold characters, Table 1; see additional file 8A–D and Figure 4). All of these genes, many of them expressed in somatic cells, are enriched in fetal wild-type ovaries compared to testis and become candidates for a role in coordinating ovarian histogenesis and/or steroidogenesis.

Novel ovarian candidate target genes that we inferred to be positively regulated by *Foxl2 *from the comparison of single and double knockout mice were assessed in *Foxl2 *transgenic embryos. All were up-regulated in XX gonads, with occasional, limited upregulation in XY gonads as well (N = 21; Figures 5B and 5C and see additional file 7). These findings confirm the ability of the microarray analysis to detect *bona fide *targets of *Foxl2*, independent of whether *Foxl2 *regulatory action is direct or indirect. The great majority of these genes had been missed in previous less complete *in vitro *microarray studies (e.g., [52]. In particular, we found limited overlap between the differentially expressed gene lists (121 of 1192 of the genes detected *in vitro *were identified *in vivo*). The expectation that genes up-regulated by *Foxl2 in vitro *would likely be down-regulated in the *Foxl2*-null ovaries (positive candidate targets), and *vice-versa *(negative candidate targets), was met only to some extent (p = 0.10, chi-square). Nevertheless, the overlapping hits included several biologically validated or promising genes, e.g., *Nr5a2, Zbtb7c, Mmp23 *and *Inhbb *(see additional file 15). In short, even though *in vitro *data included a plethora of hits that appear unrelated to the *in vivo *situation, some genes that overlap do have known or putatively important roles *in vivo*. This suggests that *in vitro *models may provide useful information in the study of gonadal development, though extensive filtering by comparison to *in vivo *data is required.

Overall, our *Foxl2 *transgenic model shows a generalized induction of ovary markers in XX embryos and a severe perturbation of testis markers in XY embryos, underlining a sex-specific action of *Foxl2*. The strongest effects of the *Foxl2 *transgene in XY embryonic gonads involved male germ cell genes and sexually dimorphic steroidogenic factors [the reduction of *Xmr, Oct4 *and P450ssc/*Cyp11a1 *to baseline levels and the strong upregulation of aromatase/*Cyp19a1 *as well as other ovarian genes, e.g., *Clstn2, Lrrc4*, and *Zbtb7c*, Figure 4]. Nevertheless, the earliest gonadal defects involving sex reveral that are observed in female steroidogenesis-deficient mice (i.e., lacking aromatase- or both estrogen receptors) occur around the time of puberty (e.g., reviewed by [16]). Thus, aromatase and estrogen action can only partly account for *Foxl2 *action, and presumably only at later stages during ovary development and maturation. The early branches of the *Foxl2 *pathway remain to be characterized more fully.

### 3) Early embryonic action of *Foxl2*

A striking result from the microarray analysis is the early time at which *Foxl2*-null ovaries showed significant anomalies, i.e., by 13.5 dpc, the time at which germ cells commit to the oocyte fate in mice. Some ovarian genes (candidate positive targets of *Foxl2*) were down-regulated and some ovarian and testis-like genes were consistently up-regulated (Figure 2 and see additional file 5). Thus, *Foxl2*-null ovaries show subtle features of partial sex reversal starting during early embryonic development. Nevertheless, levels of testis genes were low in prenatal *Foxl2*-null mouse ovaries (i.e., they increased by only 7–30% of the difference between testes and baseline signal in wild-type ovaries, by real-time quantitative PCR, Figure 2). This may explain why we previously failed to detect SOX9 protein expression at these stages [14]. In any case, the early, reproducible transcriptional dysregulation of a number of testis and ovarian genes suggests that the female sex determination program is partly impaired in embryonic ovaries that lack *Foxl2*. These subtle alterations may predispose to overt molecular sex reversal, which occurs postnatally in the supporting cell lineage of *Foxl2*-null ovaries, and is accelerated and extends to all other cell types in ovaries lacking both *Wnt4 *and *Foxl2 *(see Introduction). The data also suggest widespread antagonistic or competitive interactions between *Foxl2 *and *Wnt4 *during fetal development (see Results).

The molecular anomalies detected in *Foxl2*^-/- ^single knockout ovaries occur earlier than the earliest time of onset of histological sex reversal reported in mouse models so far (late fetal life in an *Rspo1*-null model and in the *Wnt4*^-/-^*Foxl2*^-/- ^double knock-out ovaries we have studied [17,21] and data not shown). Instead, *RSPO1 *mutations and *Foxl2 *downregulation in humans and goats, respectively, are associated with much earlier, nearly complete forms of female-to-male sex reversal [27,28].

To reconcile differences among species, one possibility infers that other genes, possibly expressed in oocytes [15,53], may be involved in female sex determination, and some might well be mouse-specific. Alternatively, as we have previously suggested, several autonomous ovarian gene pathways conjointly repress testis differentiation, but their autonomy may be more pronounced in mice than in higher mammals [16,17].

We infer several candidate anti-testis genes that are expressed independently of both *Foxl2 *and *Wnt4 *by comparing gene expression patterns associated with the partial ovary-to-testis sex reversal (as observed in the corresponding single gene knockout models) to both testis and wild-type ovary profiles. Some of these candidate anti-testis genes have been partially characterized previously – e.g., *Wnt9a *and *Irx3 *(cf. [21,54]), whereas others are novel, e.g., *Zbtb7c *(Table 4). Their function should become clearer with ablation studies to determine their interactions, with particular attention to any dependence on *Rspo1 *[21]. Genes with restricted gonadal expression also become candidates for possible involvement, via constitutive mutations, in human patients affected by non-syndromic forms of 46, XX sex reversal or premature ovarian failure.

### 4) Dosage effects in *Foxl2*^-/+ ^heterozygotes

A fourth inference from the analyses is that anomalies in *Foxl2*^+/- ^heterozygous ovaries were milder but similar to *Foxl2*-null ovaries. They included reduced expression of follicle genes, notably involved in steroidogenesis, and the transient coexpression of some fetal ovary and testis genes at levels higher than normal. These features suggest that loss of one *Foxl2 *allele might decrease the stability of ovary sex determination, and might possibly be implicated in the etiology of premature ovarian failure in heterozygous *FOXL2*^+/- ^human patients [25].

## Conclusion

The connection between the mechanisms of follicle formation and female reproductive competence in mammals has long been appreciated [3], but an involvement of embryonic anti-testis genes has only recently been suggested. Among the known anti-testis genes, *Foxl2 *has a special status because it is required in supporting cells throughout ovarian development ([14,17] and this study), and its continuous expression in postnatal primordial follicles suggests a possible role in the maintenance of female reproductive capacity [16]. However, the comprehensive analysis of available microarrays adds to the weight of evidence that additional gene pathways that may have a more limited action in time, independently promote ovarian differentiation and repress the alternative testis fate. They include at least one pathway involving *Rspo1 *and *Wnt4 *as well as novel candidate factors ([42] and see above). The identification of the full range of *independent *anti-testis genes and their most specific targets, as inferred from appropriate series of partly overlapping combinations of mutant genotypes, now becomes a focus for further investigation.

## Methods

### Mouse strains and RNAs

We previously reported the generation of mice lacking *Foxl2 *alone or in combination with *Wnt4*-null or *Kit*^*Wv *^hypomorphic alleles, as well as mice expressing a *Foxl2 *transgene [17,26]. For newborn and postnatal ovaries processed with Agilent microarrays (see below), strains were a mixed background from 129S6/SvEvTac and NIH-Swiss/BC. For 15.5 dpc *Wnt4*-null single knockout ovaries and the respective age-matched controls [47], they were a mixture of C57BL/6J and the original 129 strain. For all other ovaries processed with Affymetrix microarrays, mice were a mixture of C57BL/6J, NIH-Swiss/BC and CD1. RNA samples for each genotype and developmental stage were obtained from gonads of individual mice (biological replicates), and were processed as follows.

### RNA extraction of the samples and microarray platforms

For the Affymetrix platform, total RNA was extracted with the MELT enzymatic system (Ambion) followed by purification and linear amplification with the Pico Ovation kit (Nugen). For the Agilent Platform, total RNA was extracted by mechanical homogeneization and purified on RNeasy affinity columns.

For the Affymetrix MOE430 v.2 platform, all gonadal samples were assayed on three biological replicates, except for *Wnt4*-null mice and their age-matched controls collected at 15.5 dpc, which were studied in duplicate (that is, two mice each). Gonads from individual mice served as biological replicates; thus controls were comparable to the small numbers of double-knockout mice that were available, limited in numbers by high prenatal mortality. A step of RNA amplification was thus required. *Foxl2*-null ovaries and wild-type littermates were collected at 13.5 dpc, 16.5 dpc and on the day of birth (18 samples). Ovaries from newborn mice lacking *Foxl2 *and *Wnt4*, or *Foxl2 *and *Kit*, were compared to age-matched controls lacking *Wnt4 *or *Kit *but harboring a wild-type *Foxl2 *allele (12 samples). Testis samples were collected at 13.5 dpc and at birth. The latter were from *Wnt4*-null mice, which show partly defective testis differentiation. This was done in order to facilitate the assessment of the degree of sex reversal affecting newborn ovaries lacking both *Wnt4 *and *Foxl2 *[17].

The microarray analysis of the *Foxl2 *gene dosage effects was performed on *Foxl2*-null, *Foxl2*^+/- ^heterozygous and wild-type ovaries that were obtained in triplicate at 16.5 dpc (9 samples) or in duplicate from pools of 4–8 mice at 7 days postnatum (dpn) (4 samples). The 16.5 dpc ovary samples were hybridized on the Affymetrix MOE430 v.2 platform after RNA amplificatioon (see above), whereas the 7 dpn ovaries were assayed on the Agilent 44 k mouse developmental platform without RNA amplification.

Additional *Foxl2*-null and wild-type newborn ovaries were processed in triplicate and analyzed on the Agilent platform, mainly for cross-platform validation purposes (see below). All our microarray datasets can be downloaded from the public web site, , via the accession number GSE12989.

### Microarray data normalization

Raw Affymetrix data (.CEL) file processing and normalization was performed by the *plier *program in R (at cran.r-project.org) with an additional step of scaling to the global mean.

For data from the 44 k mouse developmental array from Agilent, after background subtraction from the Agilent analyzer raw output data, calculation of the ratio of the channels was followed by normalization of each sample values against a virtual microarray dataset by local mean regression as implemented by the *loess *program on R (span 0.01). The reference virtual dataset contained the average expression values for each probe across all samples.

### Additional available microarray datasets (available at  or )

Datasets for models of (whole-organ) gonadal dysgenesis that we have accessed from the public domain are from four distinct laboratories, as follows: *Emx2*^-/- ^(GSE10216),*Lhx8*^-/- ^(GSE7774), *Nobox*^-/- ^(GSE7775), *Foxo3*^-/- ^(GSE8249) and *Wnt4*^-/- ^kidney (GSE6934) ([42,44] and unpublished data). We processed the raw data (Affymetrix MOE430 v.2 CEL files) with *plier *on R. Normalized datasets were directly used for PCA or analyzed to obtain lists of genes that were differentially expressed in mutant vs control ovaries with the Focus software [30]. We thus selected the hits corresponding to a minimum Focus score of 4 under bootstrap-optimized FDR (see below), with the exception of the *Foxo3*^-/- ^samples. For those only a handful of genes were obtained with the same procedure. This was consistent with the inability of a published study to identify any differentially expressed genes at birth in these datasets [44]. Therefore, for the *Foxo3*^-/- ^samples, we raised the Focus score threshold for probe selection to 6 but used no FDR; we reason that the degree of concordance between the resulting gene lists and several other gene knockout models (see Results) validated the procedure. Furthermore, a strong correlation was obtained between the gene lists obtained from the *Foxo3*^-/- ^newborn ovaries and those from 7 dpn ovaries, which could be investigated with or without optimized FDR (p = 0.001; not shown). This indicates that in spite of the lack of the FDR control, the *Foxo3*^-/- ^newborn ovary gene lists were enriched in specific though possibly indirect targets of *Foxo3*. They were thus included in comparisons with the other prenatal and newborn ovary models (see Results). Gene lists for *Wnt4*^-/- ^and *Figla*^-/- ^ovaries, which were obtained with different microarray platforms, were taken from the respective reports without modification [43,45].

The datasets involving only wild-type whole-organ gonadal samples were as follows: normal ovary and testis development from 11.5 dpc to 2 dpn (GSE5334 and GSE4818) and from 11.5 to 18.5 dpc (GSE6916) from two distinct laboratories ([55] and unpublished data), and one adult ovary and testis each (as well as *Kitl *hypomorphic adult testis) from two other laboratories (GSE8249 and GSE1986, [44] and unpublished data).

One dataset was for oocytes from primordial through antral follicles (GSE3351, [37]). We used that dataset to infer lists of oocyte genes showing decreasing vs increasing expression profiles from early through growing follicles with Focus (as above), thus inferring genes expressed in fetal vs. growing *oocytes*, as given additional file 12. In order to obtain somatic cell-enriched genes, we subtracted the corresponding oocyte gene lists from the lists associated with parallel expression profiles (i.e., decreasing vs increasing, resp.) in whole-organ ovaries aged 0 through 14 dpn (GSE8249, [44]). This produced the lists of genes enriched in fetal vs postnatal *somatic *cells, in additional file 12.

Additional published microarrays on purified cells were as follows: *Sf1/Nr5a1*:GFP from ovaries and testes aged 10.5 through 13.5 dpc (E-MEXP-454, [38]) or 10.5–11.5 dpc (GSE3463, [56]), as well as *Sry*:GFP-positive supporting somatic cells aged 13.5 dpc (GSE4928, [57]). We also reanalysed a *Wnt4*^-/- ^newborn kidney microarray that was deposited but is not accompanied by publication (GSE6288).

### Principal component analysis

Several approaches are available to identify markers of biological interest from microarray data. Completely unsupervised approaches, which do not incorporate information on class identity, perform less well than their supervised counterparts, which instead use such information explicitly. Supervised algorithms include recent versions of discriminant analysis (such as "prediction analysis of microarray, PAM, [29]). The design thus favors the identification of the most strongly class-discriminatory biomarkers. However, it is not clear how the resulting informative biomarkers, besides their role in the output classifier, might be related to one another. In addition, the identification is vulnerable to any bias resulting from *a priori *definition of the classes. Therefore, a mixed unsupervised-supervised approach may represent a better solution.

Principal component analysis (PCA) is an unsupervised method for clustering and classification that, contrary to other unsupervised algorithms (such as hierarchical or k-means clustering), incorporates a statistical description of the datasets. In general, the purpose of PCA is to rotate data points into a new coordinate system, such that the majority of the variance in the data is distributed along the directions of a subset of the new axes. This facilitates visualization of significant patterns or trajectories within the data. In addition, the resulting axes are uncorrelated. Thus, each axis can represent the activity of clusters of genes showing a specific pattern (i.e., positively or negatively correlated expression patterns), or linear combinations thereof, and each pattern is uncorrelated to the others, thus possibly representing an independent biological process. PCA has its own drawbacks (e.g., interpretation of axes representing complex combinations of patterns of covariation may be challenging, and axes associated with small amounts of variance may be spurious). However, it can identify the major uncorrelated trends of variation in the data and quantify their contribution to the total variance.

Mixed unsupervised-supervised approaches to PCA have been proposed that restrict the dataset to the genes that may be considered more informative for a biological process of interest, by other statistical methods [58,59]. The resulting approach, which might be termed "calibrated PCA" (because no explicit information on predefined classes is provided), was found to perform as well as as the best fully supervised classification algorithms that are currently available [59]. Furthermore, the approach retains the ability, which is inherent in PCA, to reveal any additional unpredicted patterns that may exist within and among the known classes, thus reducing the effect of any biases at the step of class selection.

Based on these considerations, we used a general approach defining minimal "classes" as the known biological conditions, i.e., the set of replicates that were available for each combination of genotype and developmental stage under study. We obtained the lists of genes that showed the highest level of variation across such predefined classes using the Focus software ([30], setting the standard cutoff at Focus score 4; see below) and carried out standard PCA on the values for such genes in the samples of interest.

By carrying out PCA on the samples taken from *all *experiments simultaneously, the first principal components (i.e., the PCA axes accounting for the maximum variation) distinguished the experiments more than they discriminated the biological conditions; this result was obtained on rank-normalized values – that is, in spite of the use of a procedure that could help to reduce laboratory-related bias [60]. Therefore, we restricted PCA to the samples from each single experiment (the training dataset), leading to a PCA space onto which the samples from the other experiments (the test datasets) were mapped (i.e., classified). Mapping the test dataset involved the "pseudo-inverse matrix" method computed with the *svd *function in R [59]. To further reduce the effect of experimental idiosyncrasies, we obtained the developmental marker list from one dataset and trained the PCA on a distinct dataset. In particular, we used, in turn, each of the three largest developmental series available on whole-organ gonadal samples, as well as the largest dataset on somatic cells from embyonic gonads (GSE5334 and GSE4818; GSE6916; our own dataset GSE12989; and E-MEXP-454, resp., and see above). The combination of marker list and training dataset that gave the best resolution (i.e., involving the genes that were most variable across GSE5334 and GSE4818 samples as the marker list, with our dataset as the training dataset) is discussed in the main text and is reproduced in Figures 1A and 1B; further information is given in additional file 1A and 1B. The corresponding marker list is given in additional file 2B, along with a representative shorter list that showed a comparable discriminative power (at the cutoff that retained the 300 top ranking variable markers, see additional file 2A and data not shown).

We obtained the lists of genes that were most significantly associated with PC1 and PC2 of the selected PCA (see above) by dividing each PC into three equal segments between the minimum and maximum values along the coordinates, thus defining 3 classes that contained the samples mapping within the corresponding coordinates. We then used the Focus program to rank genes by the extent to which their expression levels increased or decreased through these classes (see additional files 2B–C).

### Detection and ranking of differentially expressed genes

In all cases, we applied the Focus software [30] to the entire microarray datasets, i.e., without any pre-filtering of the probes. Focus ranks genes according to the statistical distribution of contrasts (i.e., sums of weighted means) calculated as the linear transformation of four parameters (ratios and differences of expression intensities between conditions, as well as their consistency within each condition). The weights were chosen to represent arbitrary directions of change (such as, 1 and 0 in pair-wise comparisons, or 0, 1 and 2 in three-way comparisons). These values were then normalized according to the distribution of the replicates among the distinct conditions, as implemented in Focus. We used the bootstrap algorithm implemented in Focus to select hits that were associated with at least 1.1-fold mean differences, scored higher than the default "interest score" threshold of 4 and correspondingly to an "optimized" threshold value of the false discovery rate (FDR). The Focus interest score reflects the statistical distance of each probe from the mean of the probes' distribution in terms of the combined standard deviations of the four Focus parameters presented above. The bootstrap-based algorithm selects an FDR cut-off in order to simultaneously minimize the estimated proportion of false negative errors, as inferred from the observed t-test distribution of the expression values [30].

The combination of a fold-ratio with an optimized FDR is intermediate between the procedures that combine a fold-ratio with either a fixed FDR or a fixed p-value cut-off (the former is widely used but may be too stringent, the latter was recommended by the Microarray Quality Control Consortium [61]). In addition, as can be inferred from the gene lists produced for *Foxl2*-null newborn ovaries vs controls analyzed with Affymetrix versus Agilent platforms, we found that using an optimal FDR led to greater specificity (i.e., a greater fraction of consistent hits) and increased sensitivity (i.e., a larger number of total hits) than the alternative methods (not shown).

In order to detect genes that were significantly associated with the *Foxl2*-null or *Wnt4*-null ovary phenotypes, we included our wild-type and heterozygous ovary samples from all developmental stages that can be considered, phenotypically, as normal controls (see additional files 3A–B and 7A–B). This was done with the purpose of spanning the range of variation that we had observed for the normal gonadal transcriptome by PCA (see above). As a complementary approach, we also identified the genes that were shared by the three pair-wise comparisons of age-matched *Foxl2*-null and wild-type ovaries from 13.5 dpc through birth (see additional file 3C).

### Gene Ontology and KEGG analysis

We obtained the KEGG (Kyoto Encyclopedia of Genes and Genomes) and Gene Ontology classification for mouse genes from the mmu00001.keg database at ftp.genome.jp and the "GO" libraries available at  and, for Agilent probes, at  (GOENTREZID2GO function in the GO package); the analyses were performed in R (scripts available on request). The lists of differentially expressed genes between *Foxl2*- or *Wnt4*-null knockout ovaries and wild-type controls (above) were tested for any significantly different distribution across KEGG categories, i.e., by a comparing pairwise proportions using Fisher exact tests; we set a false discovery rate (FDR) threshold of 10% (see additional file 8). Similar results were obtained with GO (not shown).

### Identification of candidate genes for a role independent of *Foxl2 *and *Wnt4*

We identified the genes that showed the geatest variation according to the following profile: *Wnt4*- or *Foxl2*-null ovary > wild-type ovary > testis. For this analysis, we thus included our embryonic and testis samples in the analyses of the single knockout ovaries described above. Linear contrasts implemented in Focus helped to emphasize directions of change rather than strict pattern similarity. This three-way comparison has the advantage of reducing, in the comparison between knockout and wild-type ovaries, the relevance of both the magnitude *and the sign *of any differences or direction of change, as long as these differences were statistically small relative to the difference between ovary and testis. Because the mutant and wild-type ovaries were chosen to be morphologically and histologically closer to one another (e.g., before oocyte loss occurred in some models) than either was to testis, the risk of selecting idiosyncratic features of the mutants (e.g. *Wnt4*- or *Foxl2*-null ovary >> wild-type ovary ~testis) was expected to be low. Thus, genes were ranked according to how strongly they maintained or exacerbated a wild-type ovary-like activity relative to testis (see additional files 9A–B, combined -by boolean intersection- into additional file 9C; the top 30 genes from additional file 9C are given in Table 2).

### Real-time PCR

Using real-time PCR (TaqMan, Applied Biosystems), we assayed the same samples that were processed for the microarrays as well as additional biological samples, employing at least three biological replicates per condition. Gonads from *Foxl2 *transgenic 13.5 dpc embryos were also collected and compared to their wild-type littermates, following a heat-shock treatment to activate the transgene as previously reported [17]. Normalization was performed by scaling to expression values of the succinyl dehydrogenase A subunit, *Sdha*. Consistent results were obtained by using another gene as the scaling factor, *Gapdh*. Real-time PCR for validation of the microarray data involved the following genes (and TaqMan probes): Amh (Mm00431795_g1), Apcdd1 (Mm01257559_m1), Apoa1 (Mm00437569_m1), Aromatase (Mm00484049_m1), Asb9 (Mm00518372_m1), Bax (Mm00432050_m1), Bicd1 (Mm00802208_m1), Ccdc113 (Mm00556701_m1), Clgn (Mm00515517_m1), Cyp26b1 (Mm00558507_m1), Dax1 (Mm00431729_m1), Dhh (Mm00432820_g1), Dmrt1 (Mm00443809_m1), Dmrt3 (Mm00616649_m1), Figla1 (Mm00488823_m1), Foxl2 (Mm00843544_s1), Fst (Mm00514982_m1), Gdf9 (Mm00433565_m1), Gdnf (Mm00599849_m1), Inhbb (Mm03023992_m1), Kcnd2 (Mm00498065_m1), Mid1lip1 (Mm00471535_m1), Mmp15 (Mm00485062_m1), Mmp23 (mm00488768_m1), Nedd9 (Mm00479569_m1), Nr5a2 (Mm00446088_m1), Odz4 (Mm00446088_m1), P450ssc (Mm00490735_m1), Pcdh11x (Mm01221603_m1), Plzf (Mm01176868_m1), Ptdgs (Mm01330613_m1), Rspo1 (Mm00507077_m1), Sgms2 (Mm00512327_m1), Sox9 (Mm00448840_m1), Sycp3 (Mm00488519_m1), Wnt4(ex1–2) (Mm01194003_m1), Xmr (Mm00784689_s1), Zbtb7c (Mm00520231_m1). All results were consistent with the microarray data, with the partial exception for Mid1lip1 and Pcdh11x (Figure 2 and data not shown). The latter may correspond to alternative isoforms differentially recognized by microarray vs PCR assays.

Real-time PCR for the study of embryonic gonads from the transgenic mice (Figure 4 and additional file 11) involved the following genes (and TaqMan probes): Akrc14 (Mm00506336_m1), Amh (Mm00431795_g1), Clstn2 (Mm00502574_m1), Cyp19a1 (Mm00484049_m1), Dax1 (Mm00431729_m1), Dhh (Mm00432820_g1), Dmc1h (Mm00494485_m1), Foxp1 (Mm00474845_m1), Fst (Mm00514982_m1), Gpc4 (Mm00515035_m1), Grip1 (Mm00503844_m1), Inhbb (Mm03023992_m1), Kitl (Mm00442972_m1), Lrrc4 (Mm02527970_s1), Mllt1 (Mm00452080_m1), Msx1 (Mm00440330_m1), Oct4 (Mm00658129_gH), Odz4 (Mm00496266_m1), P450ssc (Mm00490735_m1), Plxnc1 (Mm00450687_m1), Rspo1 (Mm00507077_m1), Sgms2 (Mm00512327_m1), Sox9 (Mm00448840_m1), Sycp3 (Mm00488519_m1), Tcf4 (Mm00443198_m1), Wnt4 (Mm01194003_m1), Xmr (Mm00784689_s1), Zbtb7c (Mm00520231_m1).

## Authors' contributions

JEGO, EP, SO, JK, YP, and CO performed the experiments. TN, MU, AC, SWC and CO contributed essential material or new analytical tools. AF, DS and CO designed and supervised the project and wrote the manuscript. All authors read and approved the final manuscript.

## Supplementary Material

Additional file 1 **The PCA plot from main text **Figure 2** (x-axis: PC1; y-axis: PC2) in which the gonadal samples are coded to highlight additional features**. A) distinct colored symbols and shapes indicate distinct experimental source (detailed next), sex, genotype, and developmental stage (detailed in the margins). "E" indicates embryonic day; "P" indicates postnatal day. B) color coding now is for the datasets of origin; distinct colors thus represent distinct experiments performed by different laboratories (with the number of samples per dataset that is given in brackets; see Methods). Shape-coding, as in A. Click here for file

Additional file 2 **Developmental markers for the PCA, thus including all the markers identified as significant by Focus analysis**. The list in A (top 6455 probes) was used for the main figures; the lists of the genes that were most significantly associated with the first and second principal components (PC1 and PC2) are given in B and C, resp. Click here for file

Additional file 3 **Genes that were down- (A, C) or up-regulated (B, D) in *Foxl2*-null ovaries aged 13.5 dpc through birth compared to age-matched controls**. Scores (second column from left) are standard deviations of four parameters for differential expression obtained with the Focus program (see text). A-B: overall ("multiwise") comparison of three developmental stages using linear contrasts with Focus (i.e., 6 genotype × stage conditions). C-D: overlap of the gene lists obtained by three separate pairwise analyses each confined to age-matched conditions (i.e., each developmental stage was analyzed separately; Focus scores were averaged over the three analyses). Click here for file

Additional file 4 **Genes that are consistently down- (C) or up- (D) regulated in all three pathological conditions involving *Foxl2 *loss that we have tested**. A-B. Genes that are down- or up- regulated (A-B, resp.) in double knockout ovaries lacking *Foxl2 *and either *Wnt4 *or *Kit *relative to controls. C-D: genes shared between such two models of double knockout ovaries and the ovaries lacking *Foxl2 *alone (from additional files 3A–B). Click here for file

Additional file 5 **Microarray gene expression profiles of the genes that were assayed by real-time PCR in Main **Figure 2. For each gene, normalized expression intensities (y-axis) are represented as a fraction of the maximum mean value observed (the latter being set to 100). Click here for file

Additional file 6 **Genes that were differentially expressed in single knockout ovaries lacking genes different from *Foxl2***. A-B, genes that were down- or up-regulated in *Wnt4*-null ovaries aged 15.5 dpc relative to controls aged 13.5 dpc through birth. C-J, genes that were down- (C, E, G, I) or up- (D, F, H, J) regulated in *Lhx8*- (C, D), *Nobox*- (E, F), *Foxo3*-null (G, H) newborn ovaries or *Emx2*^-/- ^(I, J) 10.5 dpc bipotential gonads relative to age-matched controls. Click here for file

Additional file 7 **Overlap between the lists of *Foxl2*- or *Wnt4*-dependent genes**. A, downregulated, B, upregulated in both single knockout ovaries relative to controls, as determined from additional files 3A–B and 6C–D. Click here for file

Additional file 8 **KEGG classification of the gene lists relevant to **Table 3. The gene lists are derived from additional files 3A–B (down and up in *Foxl2*-null fetal ovaries or in *Wnt4*-null fetal ovaries relative to wild-type controls (from additional files 3A–B and 6A–B). A-B, *Foxl2*-null ovaries vs wild-type; C-D, *Wnt4*-null ovaries vs wild-type. All KEGG categories with p-value less than 0.1 by Fisher exact test (comparison of proportions) are included. Columns from left to right: Pathway name, numbers of genes for the knockout and for the wild-type ovaries, p-value, proportions for the knockout and for the wild-type ovaries, and finally two standard gene identifications. The 10% FDR threshold p-value is 0.0077 for A-B, and 0.0054 for C-D. Click here for file

Additional file 9 **Lists of anti-testis genes that were inferred to have an action independent of *Foxl2 *(A), *Wnt4 *(B) or both (C)**. comparison of knockout ovaries to both ovary and testis (see text for details on the approach). Click here for file

Additional file 10 **Real-time PCR showing *Foxl2 *transcript levels in *Foxl2 *transgenic mouse embryonic gonads**. XX or XY trangenic embryos were compared to wildtype littermates. Click here for file

Additional file 11 **Real-time PCR validation in *Foxl2 *transgenic mouse embryos and wildtype littermates**. This panel includes *Foxl2*-dependent genes (based on knockout models, see text) that are induced at higher levels in *Foxl2 *transgenic XX gonads and are either weakly induced (*Plxnc1, Tcf4, Odz4*) or not induced (*Gpc4, Foxp1, Mllt3, Akr1c14*) in *Foxl2 *transgenic XY gonads. Click here for file

Additional file 12 **Oocyte and somatic cell-enriched gene lists represented by the corresponding probes in the Affymetrix MOE430 v.2 (A) and Agilent mouse developmental (B) platforms**. These lists were used for GSEA analysis presented in additional file 13. They correspond to the top-scoring hits from additional microarray analyses (see text) with an arbitrary cut-off at the 50^th ^or 100^th ^rank (labeled as "Top50" and "Top100"). Additional labels (see Methods for details): OvFetSoma, enriched in fetal ovary somatic cells relative to bipotential gonad and to testis (*Sf1*-positive cells); FetalOo, enriched in newborn oocytes relative to postnatal growing oocytes; OoGrow, enriched in postnatal growing oocytes relative to newborn oocytes; SomaGrow, enriched in postnatal growing somatic cells by an indirect analysis, i.e., enriched in postnatal versus newborn whole-organ ovary preparation and excluded from the "OoGrow" markers; "testis determination markers", genes known to be involved in gonadal XY sex reversal when inactivated. Click here for file

Additional file 13 **GSEA plots for the study of *Foxl2*-gene dosage effects**. Any deviation from random of the distribution of 9 lists of marker genes given in additional file 12 is tested between the following conditions. Labels at the top of each panel correspond to the acronyms that denote the lists of additional file 12. A blue-red gradient along the x-axis indicates increasing differential expression toward either condition under comparison. Values for the relevant statistical parameter are shown on the y-axis by a green line. Thus, the highest vs lowest peak of this curve relative to a horizontal black line (baseline) provide estimates for the degree of enrichment toward either the "red" or the "blue" condition, respectively. A, *Foxl2*-null (E16ko, red) vs *Foxl2*^+/- ^heterozygous ovaries at E16.5 dpc (E16het, blue); B, E16het (red) vs *Foxl2*^+/+ ^wild-type ovaries at E16.5 dpc (E16wt, blue); C, E16ko (blue) vs E16wt (red); D, *Foxl2*-null (P7ko, red) vs *Foxl2*^+/- ^ovaries at 7 dpn (P7het, blue); E, P7het (red) vs *Foxl2*^+/+ ^wild-type ovaries at 7 dpn (P7wt, blue); F, P7ko (red) vs P7wt (blue). Click here for file

Additional file 14 **Representative gene expression levels (microarray) that illustrate *Foxl2*-gene dosage effects in newborn (P0) or 7 dpn ovaries (P7)**. Top panel: somatic cell genes; bottom panel: oocyte genes. Note that P7 heterozygous ovaries (2^nd ^group of bars from right) express high levels of oocyte genes and low levels of somatic genes compared to age-matched wild-type ovaries (right-most group of bars). Click here for file

Additional file 15 **Comparison of our *in vivo *gene list from *Foxl2*-null ovaries with a published *in vitro *gene list**. Our list is from additional files 3A–B. The *in vitro *gene list is from an ovarian cell line transiently transfected with *Foxl2 *(see text). Click here for file
