# Supplementary material for: Foxl2 functions in sex determination and histogenesis throughout mouse ovary development
Source: BMC Dev Biol. 2009 Jun 18;9:36. doi: 10.1186/1471-213X-9-36 (PMC2711087; doi:10.1186/1471-213X-9-36)
Supplement: Additional file 1 — The PCA plot from main text Figure 2 (x-axis: PC1; y-axis: PC2) in which the gonadal samples are coded to highlight additional features. A) distinct colored symbols and shapes indicate distinct experimental source (detailed next), sex, genotype, and developmental stage (detailed in the margins). "E" indicates embryonic day; "P" indicates postnatal day. B) color coding now is for the datasets of origin; distinct colors thus represent distinct experiments performed by different laboratories (with the number of samples per dataset that is given in brackets; see Methods). Shape-coding, as in A. [file 1471-213X-9-36-S1.pdf]

A. PCA plot labeled according to multiple criteria.

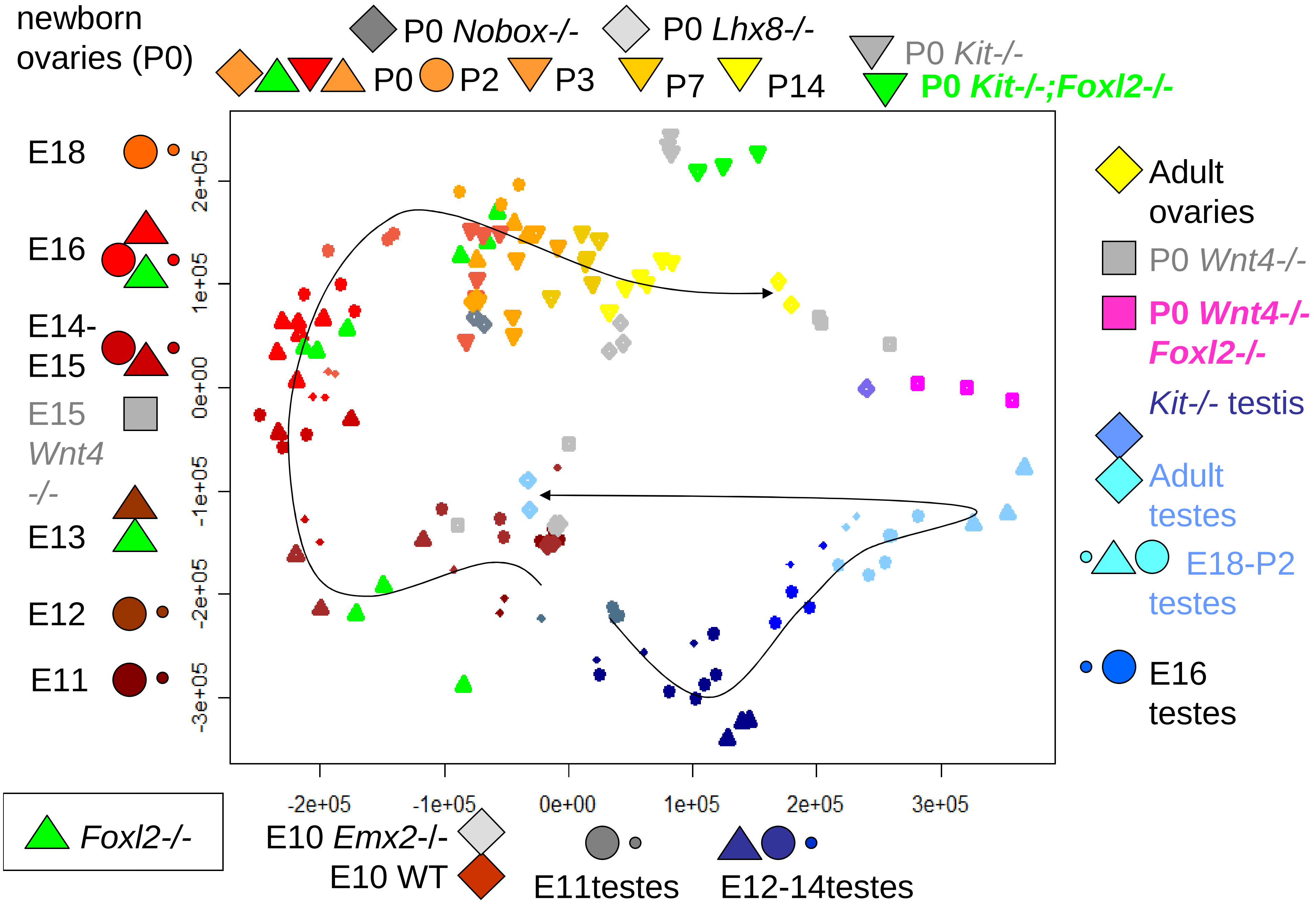

B. PCA plot labeled according to the source microarray datasets.

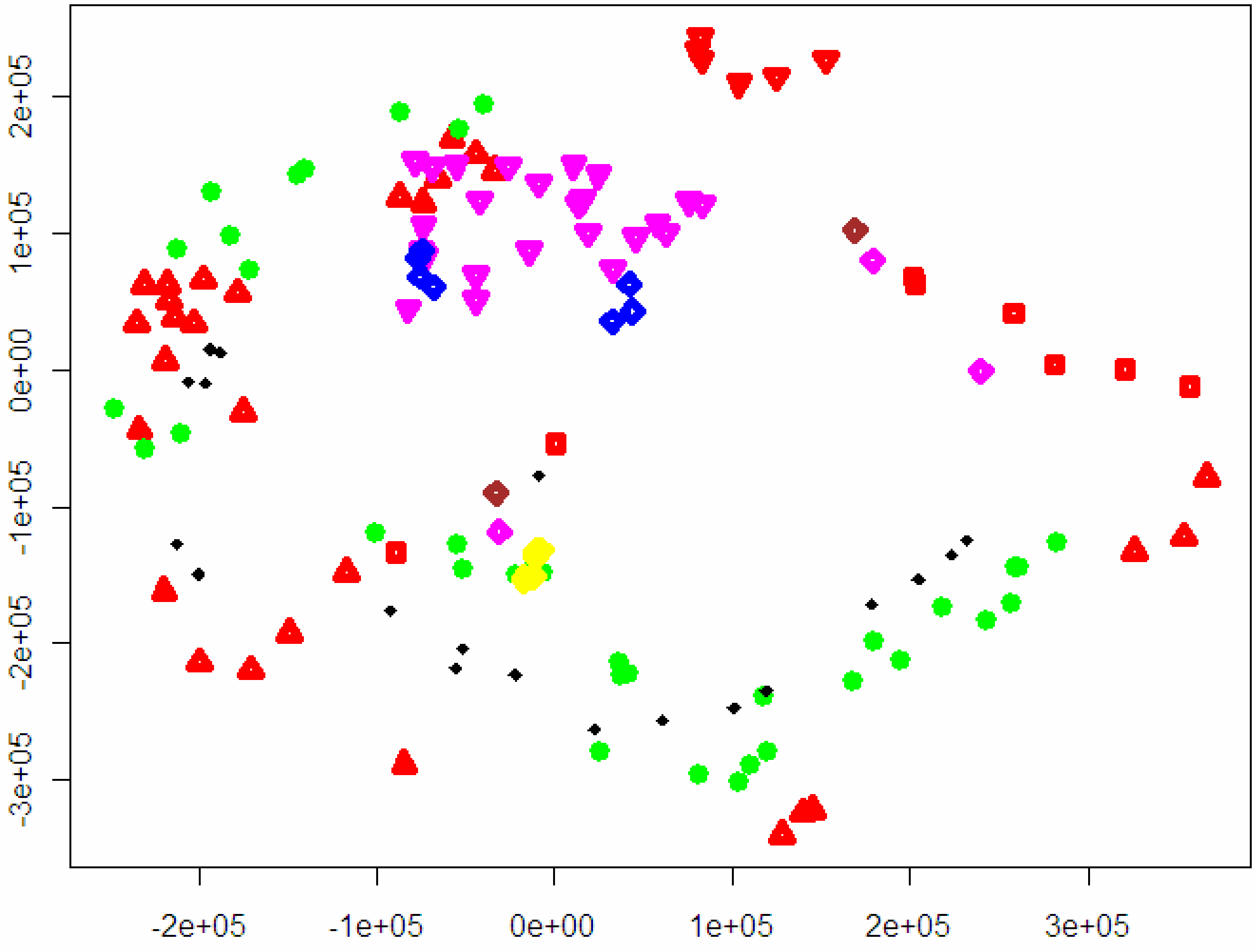

***Color coding*:**
Our dataset
(**GSE12989** , N=43)
GSE8249 (N=27)
GSE5334 (N=18)
GSE4818 (N=18)
GSE6916 (N=20)
GSE1986 (N=2)
GSE7774-5 (N=7)
GSE10216 (N=6)
