# Supplementary material for: Foxl2 functions in sex determination and histogenesis throughout mouse ovary development
Source: BMC Dev Biol. 2009 Jun 18;9:36. doi: 10.1186/1471-213X-9-36 (PMC2711087; doi:10.1186/1471-213X-9-36)
Supplement: Additional file 5 — Microarray gene expression profiles of the genes that were assayed by real-time PCR in Main Figure 2. For each gene, normalized expression intensities (y-axis) are represented as a fraction of the maximum mean value observed (the latter being set to 100). [file 1471-213X-9-36-S5.pdf]

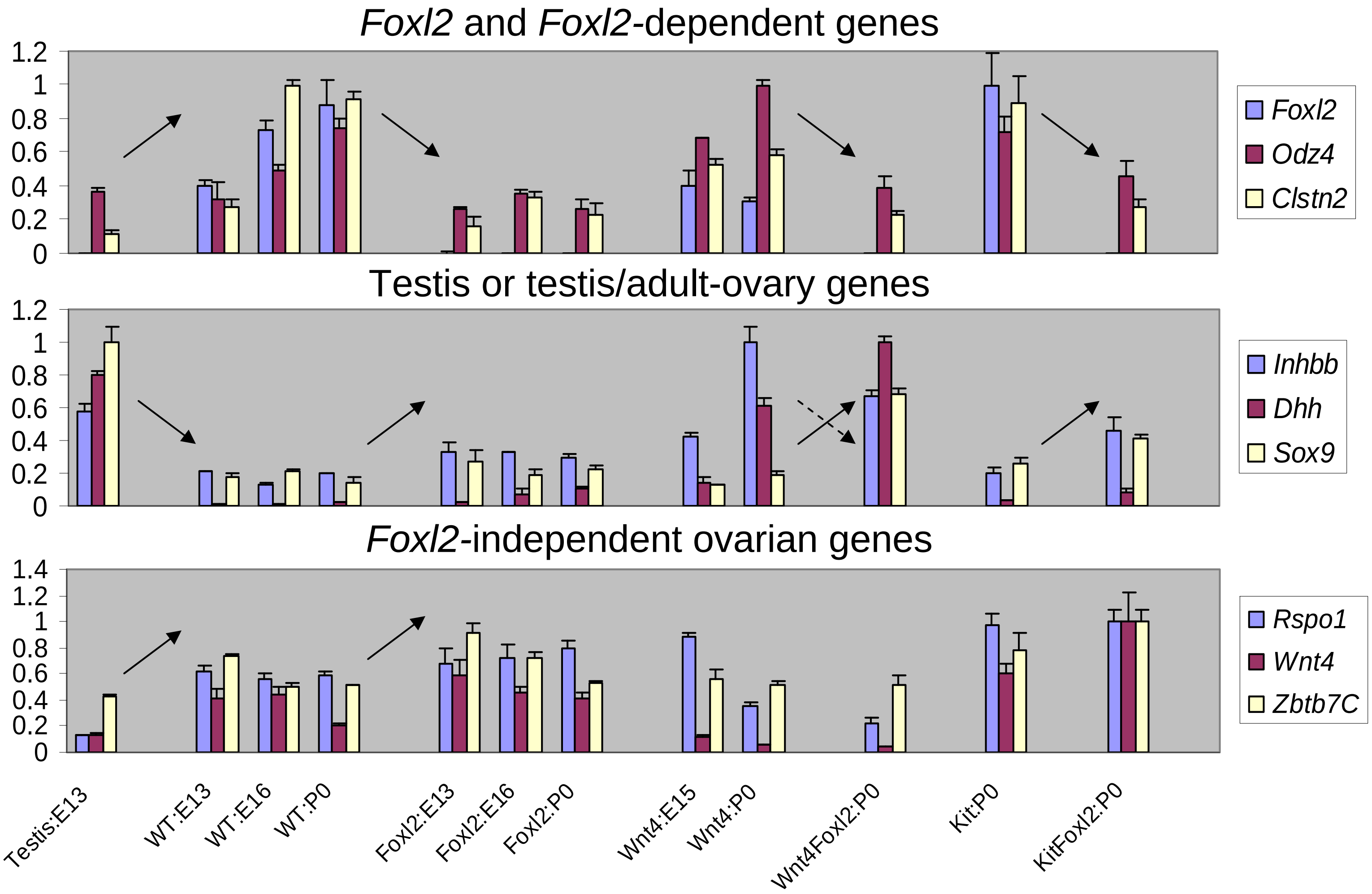

Foxl2 and Foxl2-dependent genes
1.2
1
0.8
0.6
0.4
0.2
0
Foxl2
Odz4
Clstn2
Testis or testis/adult-ovary genes
1.2
1
0.8
0.6
0.4
0.2
0
Inhbb
Dhh
Sox9
Foxl2-independent ovarian genes
1.4
1.2
1
0.8
0.6
0.4
0.2
0
Rspo1
Wnt4
Zbtb7C
Testis:E13
WT:E13
WT:E16
WT:P0
Foxl2:E13
Foxl2:E16
Foxl2:P0
Wnt4:E15
Wnt4:P0
Wnt4Foxl2:P0
Kit:P0
KitFoxl2:P0
