# Supplementary figures and images for: Foxl2 functions in sex determination and histogenesis throughout mouse ovary development
Source: BMC Dev Biol. 2009 Jun 18;9:36. doi: 10.1186/1471-213X-9-36 (PMC2711087; doi:10.1186/1471-213X-9-36)

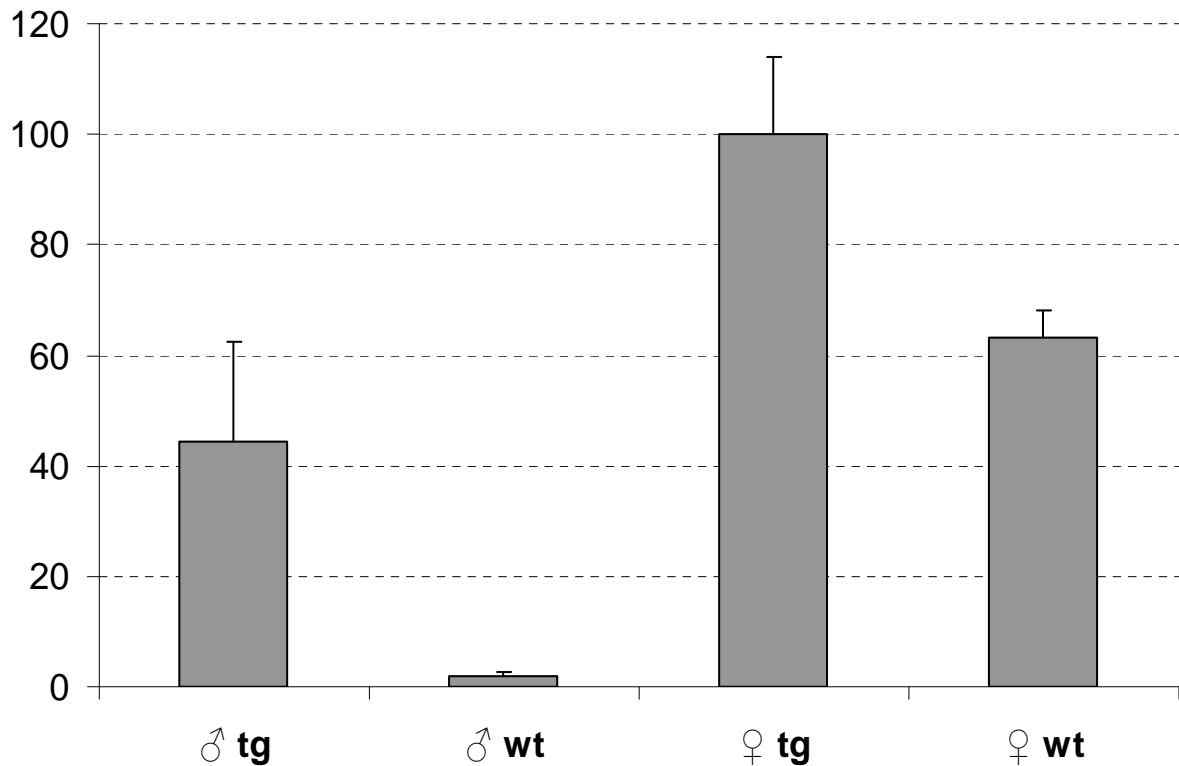

Supplement: Additional file 10 — Real-time PCR showing Foxl2 transcript levels in Foxl2 transgenic mouse embryonic gonads. XX or XY trangenic embryos were compared to wildtype littermates. [file 1471-213X-9-36-S10.pdf]

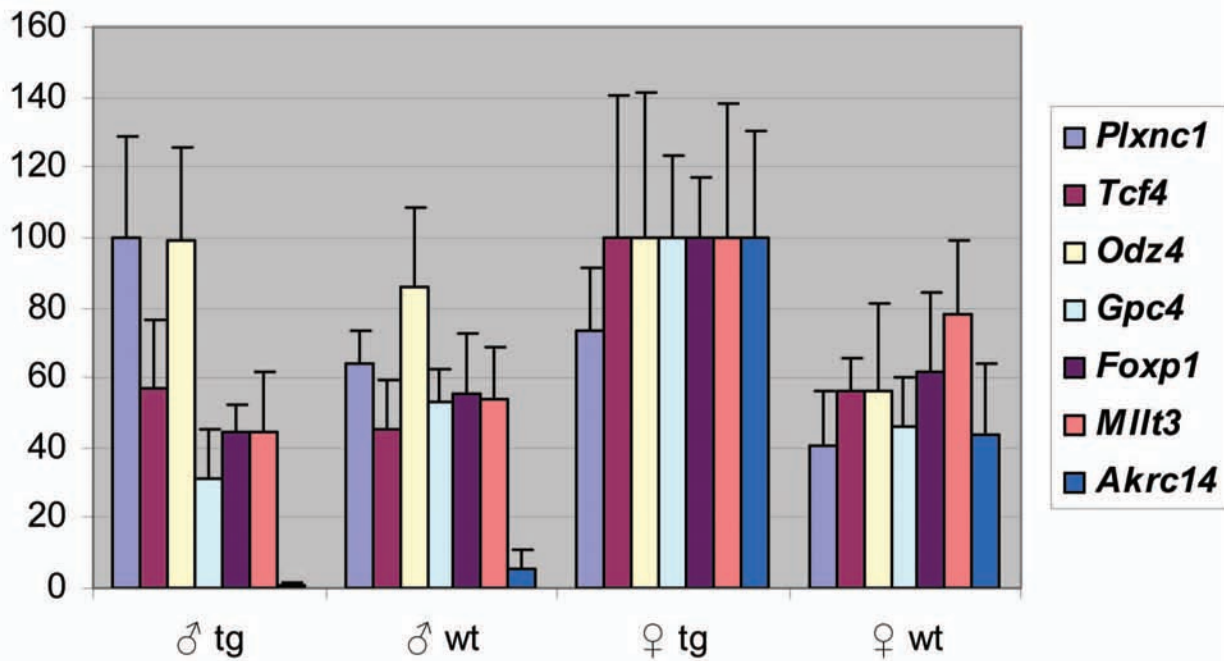

Supplement: Additional file 11 — Real-time PCR validation in Foxl2 transgenic mouse embryos and wildtype littermates. This panel includes Foxl2-dependent genes (based on knockout models, see text) that are induced at higher levels in Foxl2 transgenic XX gonads and are either weakly induced (Plxnc1, Tcf4, Odz4) or not induced (Gpc4, Foxp1, Mllt3, Akr1c14) in Foxl2 transgenic XY gonads. [file 1471-213X-9-36-S11.pdf]

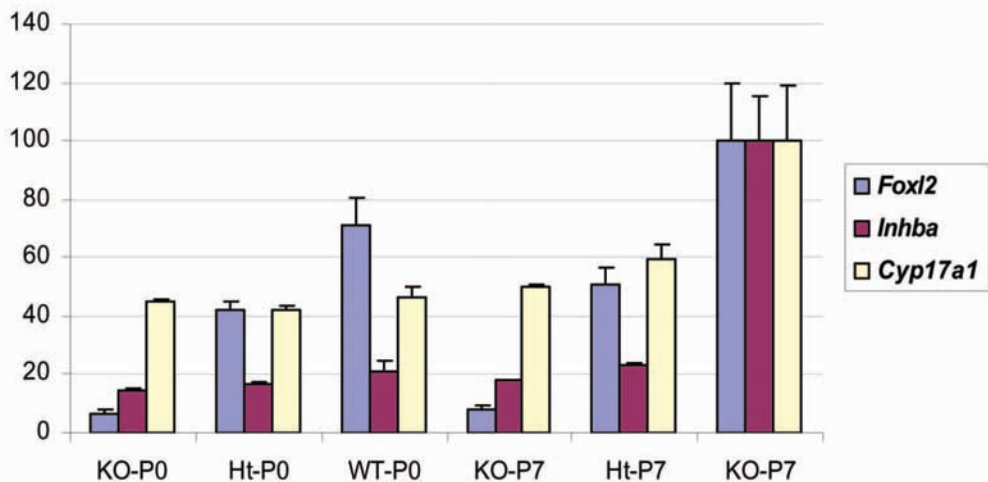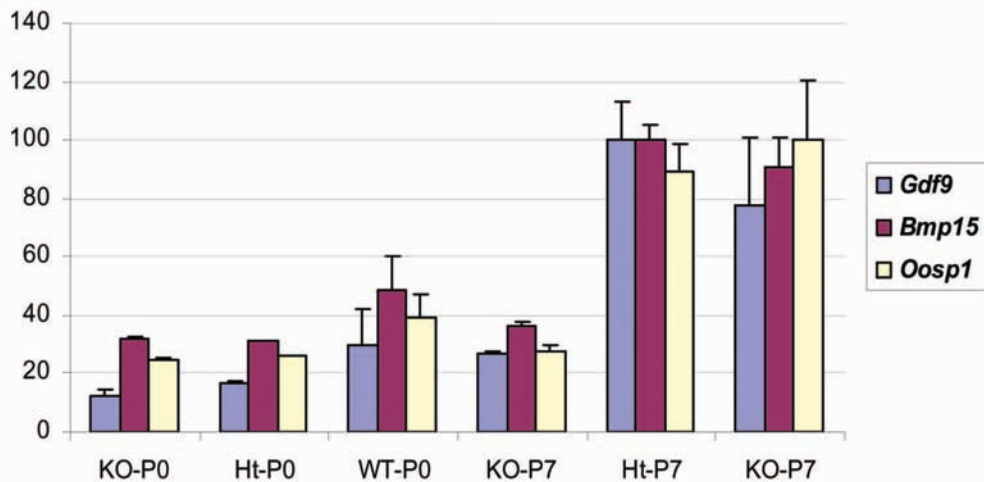

Supplement: Additional file 14 — Representative gene expression levels (microarray) that illustrate Foxl2-gene dosage effects in newborn (P0) or 7 dpn ovaries (P7). Top panel: somatic cell genes; bottom panel: oocyte genes. Note that P7 heterozygous ovaries (2nd group of bars from right) express high levels of oocyte genes and low levels of somatic genes compared to age-matched wild-type ovaries (right-most group of bars). [file 1471-213X-9-36-S14.pdf]
