# Supplementary material for: Foxl2 functions in sex determination and histogenesis throughout mouse ovary development
Source: BMC Dev Biol. 2009 Jun 18;9:36. doi: 10.1186/1471-213X-9-36 (PMC2711087; doi:10.1186/1471-213X-9-36)

A – GSEA plots for *Foxl2*-null (E16ko) vs *Foxl2+/-* heterozygous ovaries at 16.5 dpc (E16het)

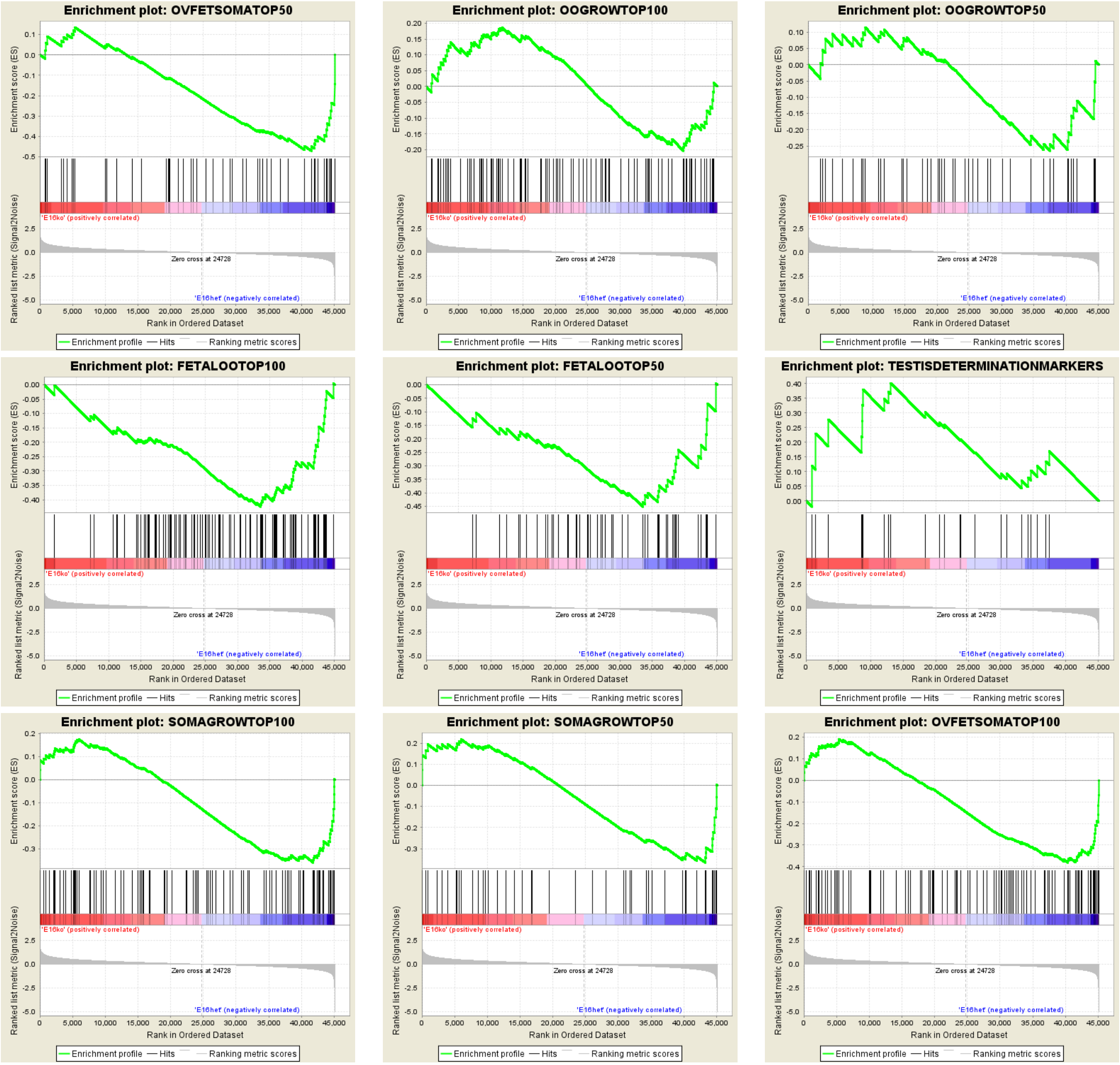

B – GSEA plots for *Foxl2+/+* wild-type (E16wt) vs *Foxl2+/-* heterozygous ovaries at 16.5 dpc (E16het)

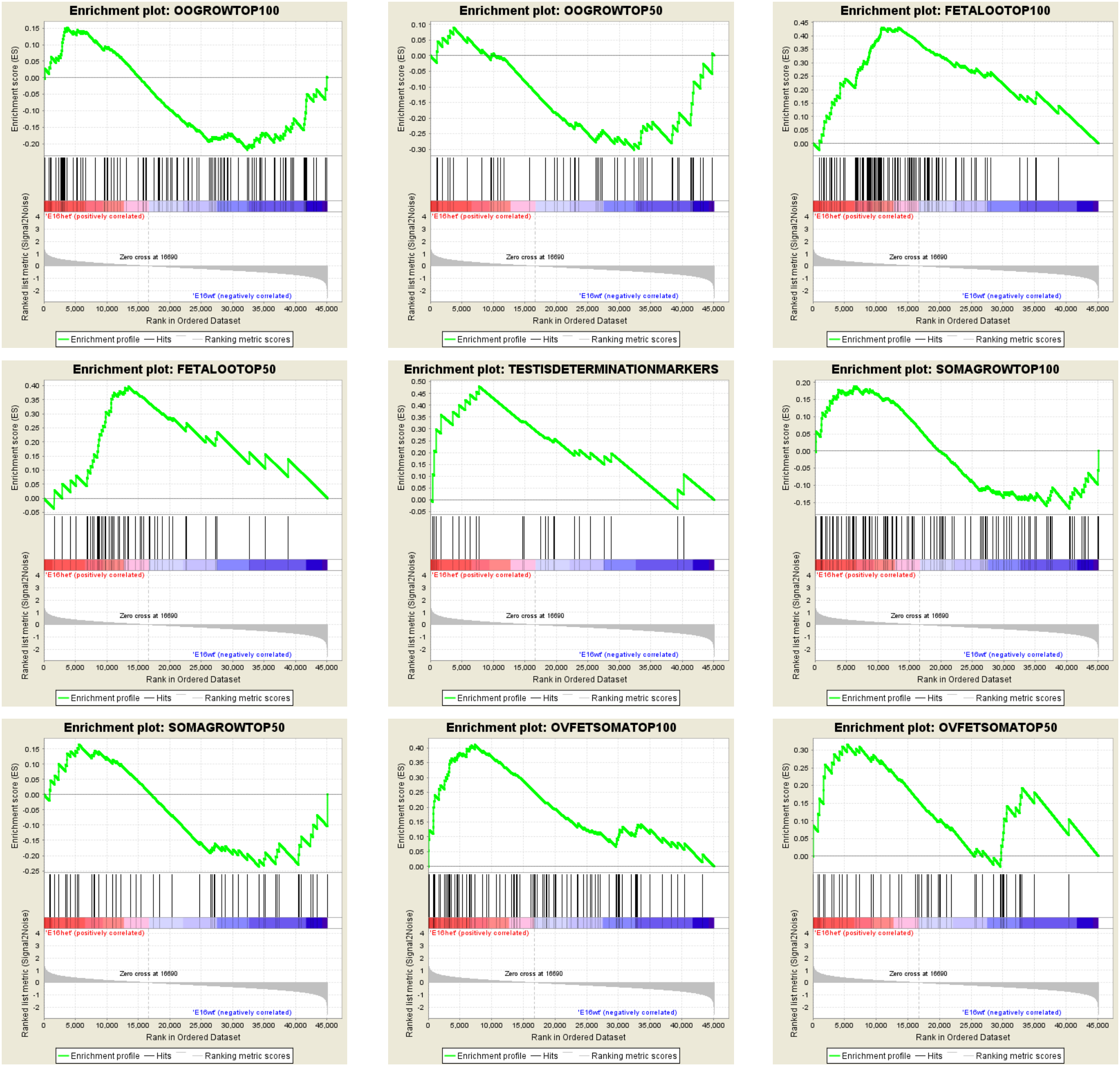

C – GSEA plots for *Foxl2*-null ovaries (E16ko) vs *Foxl2*+/+ wild-type ovaries at 16.5 dpc (E16wt).

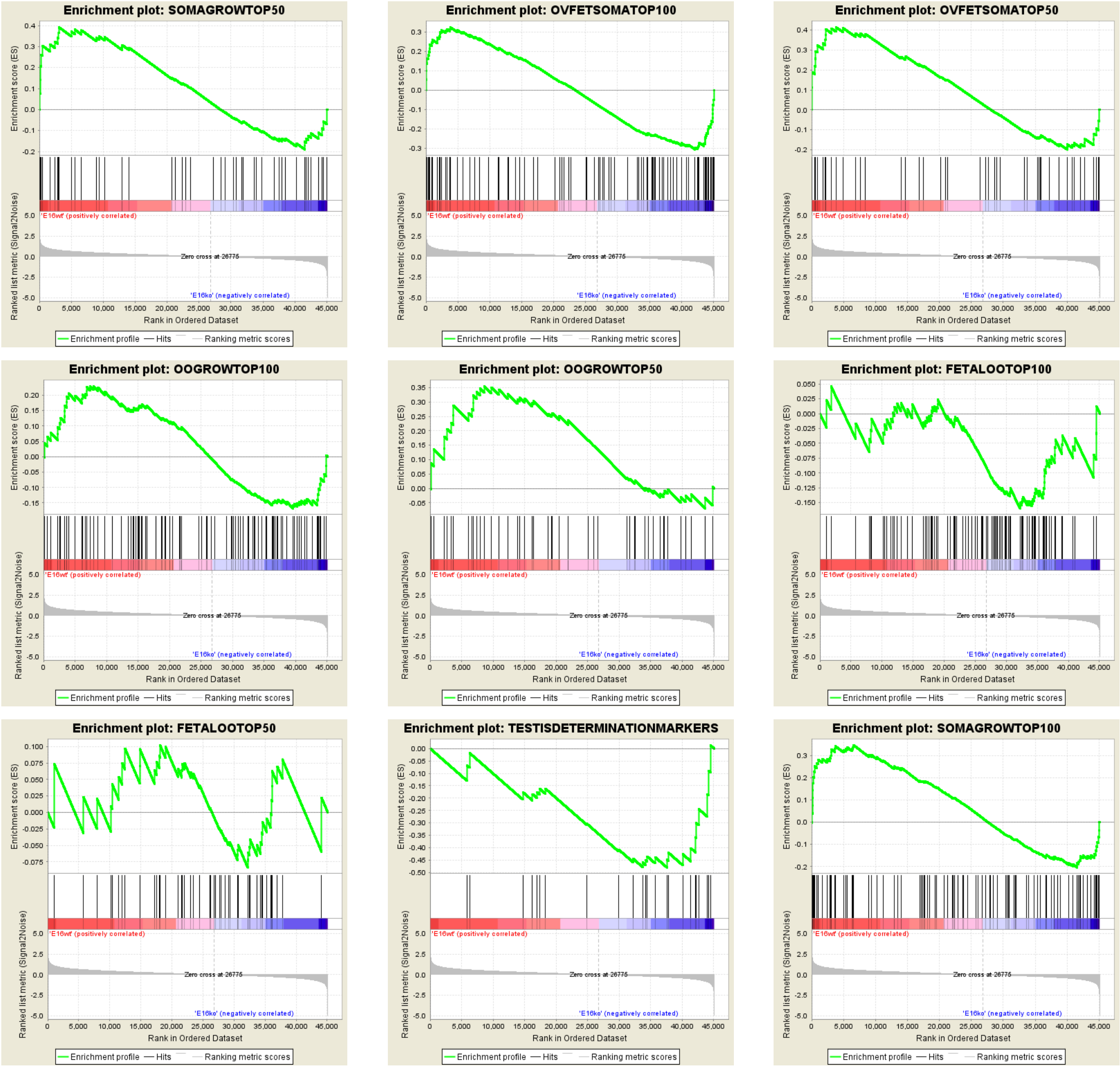

D – GSEA plots for *Foxl2*-null (P7ko) vs *Foxl2+/-* heterozygous ovaries at 7 dpn (P7het)

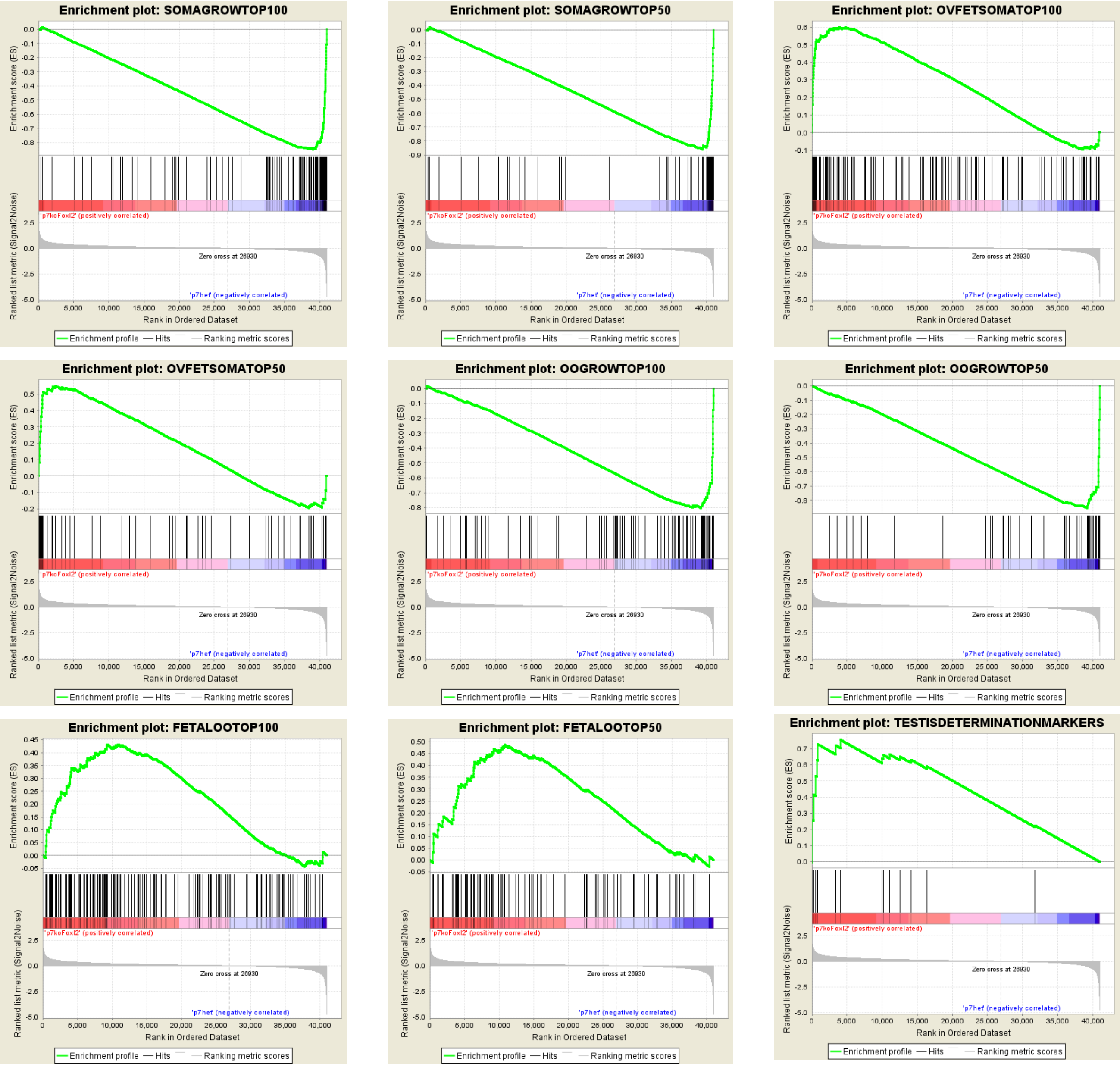

E – GSEA plots for *Foxl2+/+* wild-type (P7wt) vs *Foxl2+/-* heterozygous ovaries at 7 dpn (P7het)

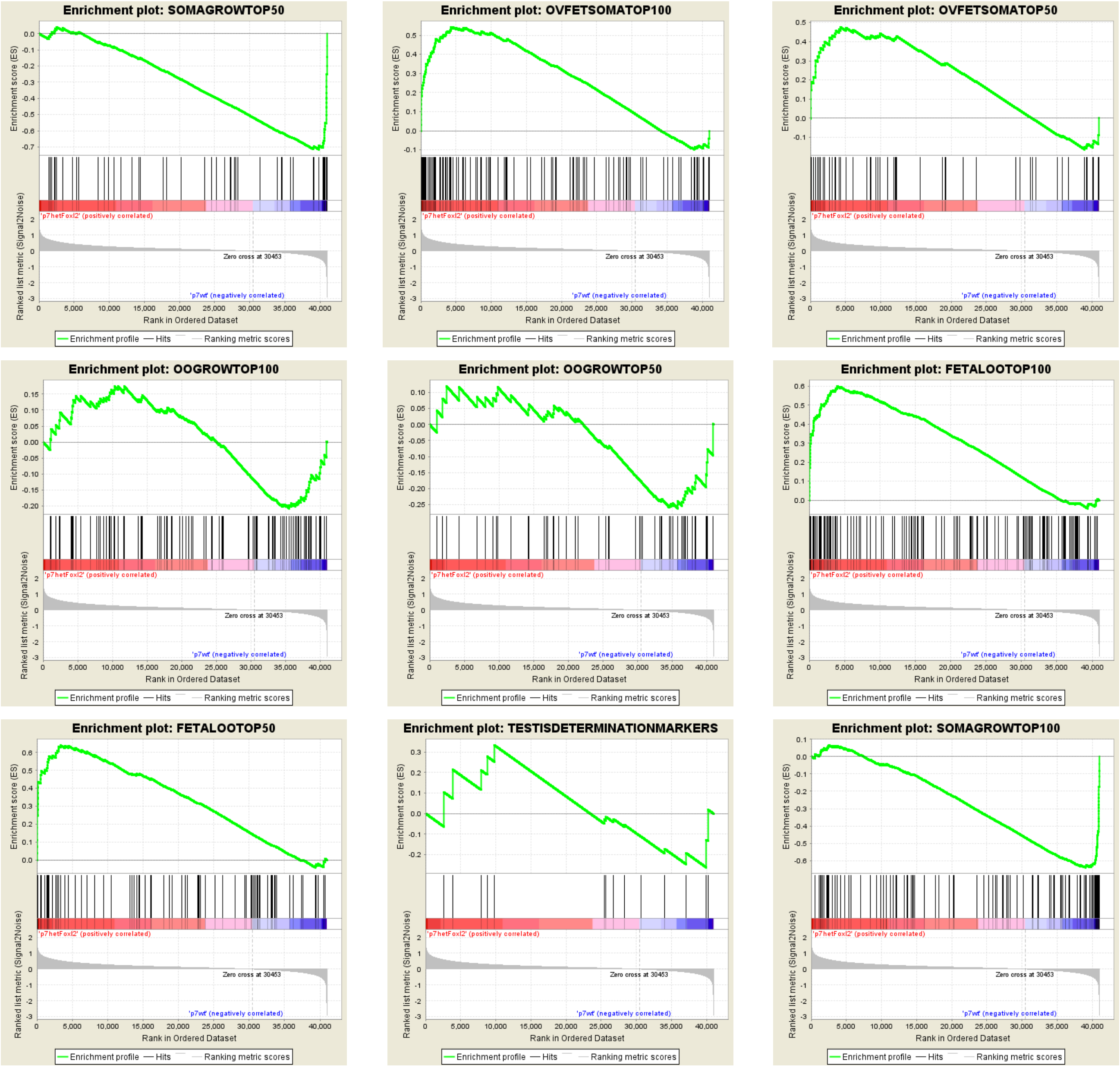

F – GSEA plots for *Foxl2*-null ovaries (P7ko) vs *Foxl2*+/+ wild-type ovaries at 7 dpn (P7wt).

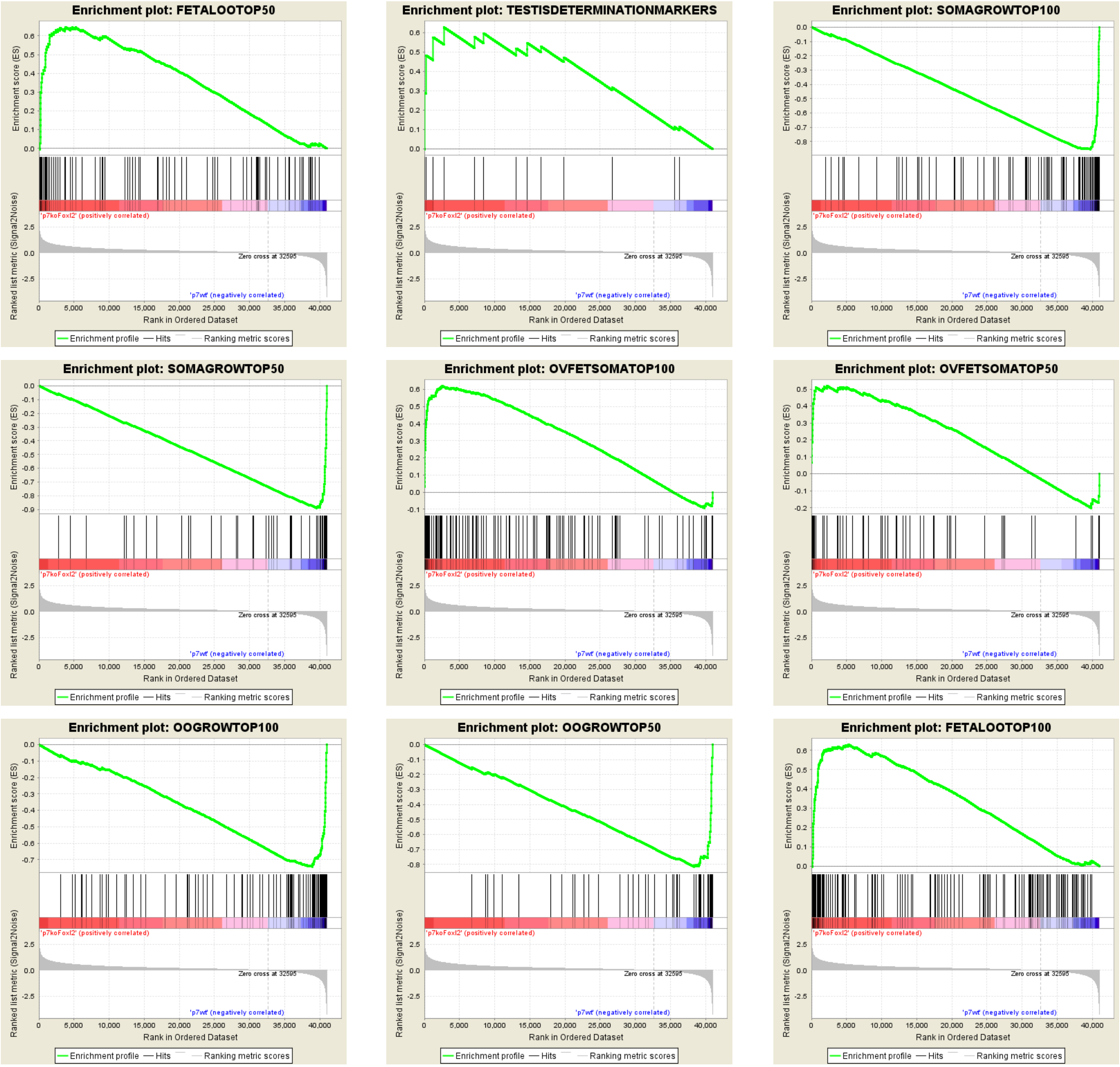

Supplement: Additional file 13 — GSEA plots for the study of Foxl2-gene dosage effects. Any deviation from random of the distribution of 9 lists of marker genes given in additional file 12 is tested between the following conditions. Labels at the top of each panel correspond to the acronyms that denote the lists of additional file 12. A blue-red gradient along the x-axis indicates increasing differential expression toward either condition under comparison. Values for the relevant statistical parameter are shown on the y-axis by a green line. Thus, the highest vs lowest peak of this curve relative to a horizontal black line (baseline) provide estimates for the degree of enrichment toward either the "red" or the "blue" condition, respectively. A, Foxl2-null (E16ko, red) vs Foxl2+/- heterozygous ovaries at E16.5 dpc (E16het, blue); B, E16het (red) vs Foxl2+/+ wild-type ovaries at E16.5 dpc (E16wt, blue); C, E16ko (blue) vs E16wt (red); D, Foxl2-null (P7ko, red) vs Foxl2+/- ovaries at 7 dpn (P7het, blue); E, P7het (red) vs Foxl2+/+ wild-type ovaries at 7 dpn (P7wt, blue); F, P7ko (red) vs P7wt (blue). [file 1471-213X-9-36-S13.pdf]
